# Single-cell DNA and RNA sequencing reveals the dynamics of intra-tumor heterogeneity in a colorectal cancer model

**DOI:** 10.1186/s12915-021-01147-5

**Published:** 2021-09-21

**Authors:** Hanako Ono, Yasuhito Arai, Eisaku Furukawa, Daichi Narushima, Tetsuya Matsuura, Hiromi Nakamura, Daisuke Shiokawa, Momoko Nagai, Toshio Imai, Koshi Mimori, Koji Okamoto, Yoshitaka Hippo, Tatsuhiro Shibata, Mamoru Kato

**Affiliations:** 1 Division of Bioinformatics, National Cancer Center Research Institute, 5-1-1 Tsukiji, Chuo-ku, Tokyo, 104-0045 Japan; 2 Division of Cancer Genomics, National Cancer Center Research Institute, 5-1-1 Tsukiji, Chuo-ku, Tokyo, 104-0045 Japan; 3 Department of Animal Experimentation, National Cancer Center Research Institute, 5-1-1 Tsukiji, Chuo-ku, Tokyo, 104-0045 Japan; 4 Division of Cancer Differentiation, National Cancer Center Research Institute, 5-1-1 Tsukiji, Chuo-ku, Tokyo, 104-0045 Japan; 5 Department of Surgery, Kyushu University Beppu Hospital, 101 Hasamamachiidaigaoka, Yufu, Oita 879-5593 Japan; 6 Division of Biochemistry and Molecular Carcinogenesis, Chiba Cancer Center Research Institute, 666-2 Nitona-cho, Chiba Chuo-ku, Chiba, 260-8717 Japan; 7 Laboratory of Molecular Medicine, Human Genome Center, The Institute of Medical Science, The University of Tokyo, 4-6-1 Shiroganedai, Minato-ku, Tokyo, 108-8639 Japan

**Keywords:** Single-cell sequencing, Cancer genomics, Colorectal cancer, Tumorigenesis, Intra-tumor heterogeneity

## Abstract

**Background:**

Intra-tumor heterogeneity (ITH) encompasses cellular differences in tumors and is related to clinical outcomes such as drug resistance. However, little is known about the dynamics of ITH, owing to the lack of time-series analysis at the single-cell level. Mouse models that recapitulate cancer development are useful for controlled serial time sampling.

**Results:**

We performed single-cell exome and transcriptome sequencing of 200 cells to investigate how ITH is generated in a mouse colorectal cancer model. In the model, a single normal intestinal cell is grown into organoids that mimic the intestinal crypt structure. Upon RNAi-mediated downregulation of a tumor suppressor gene *APC*, the transduced organoids were serially transplanted into mice to allow exposure to in vivo microenvironments, which play relevant roles in cancer development. The ITH of the transcriptome increased after the transplantation, while that of the exome decreased. Mutations generated during organoid culture did not greatly change at the bulk-cell level upon the transplantation. The RNA ITH increase was due to the emergence of new transcriptional subpopulations. In contrast to the initial cells expressing mesenchymal-marker genes, new subpopulations repressed these genes after the transplantation. Analyses of colorectal cancer data from The Cancer Genome Atlas revealed a high proportion of metastatic cases in human subjects with expression patterns similar to the new cell subpopulations in mouse. These results suggest that the birth of transcriptional subpopulations may be a key for adaptation to drastic micro-environmental changes when cancer cells have sufficient genetic alterations at later tumor stages.

**Conclusions:**

This study revealed an evolutionary dynamics of single-cell RNA and DNA heterogeneity in tumor progression, giving insights into the mesenchymal-epithelial transformation of tumor cells at metastasis in colorectal cancer.

**Supplementary Information:**

The online version contains supplementary material available at 10.1186/s12915-021-01147-5.

## Background

It is well established that cancer is pathologically composed of different types of cells [1]; however, intra-tumor heterogeneity (ITH) has only been recently addressed at the genomic level [2]. ITH is clinically important. For example, elevated copy-number heterogeneity is related to an increased risk of recurrence or death in non-small-cell lung cancer [3]. High levels of ITH ultimately provide the seeds for the emergence of anticancer drug resistance [4]. High levels of genetically characterized heterogeneity in Barrett’s esophagus are associated with incidence of esophageal adenocarcinoma [5].

ITH essentially stands for the cellular differences in tumor tissue arising from genetic changes, called clonal evolution, or non-genetic changes, such as cancer stem cells and transcriptional responses to the environment. In clonal evolution, as in Darwinian evolution, cancer cells with advantageous genetic mutations evolve into different types of cancer cells [6]. In contrast, cancer stem cells, like normal stem cells, produce a variety of differentiated daughter cells that constitute phenotypically distinct cancer cells without genetic differences through epigenetic and the resultant transcriptional mechanisms [7, 8].

A flood of studies have addressed ITH through the variant allele frequencies (VAFs) of tumor cells in bulk, which are calculated from sequence reads with variants identified through next-generation sequencing (reviewed in [2, 9]). In this bulk-cell sequencing approach, the presence of minor clones is often reflected on lower VAFs than the VAF of the major clone [10]. However, this bulk-cell DNA sequencing approach is limited in revealing genetic ITH because it only infers the presence of clones, not directly observing individual cells. In addition, the bulk-cell approach is generally not suitable to resolve transcriptomic ITH, where transcript mixtures from different cells are sequenced.

Single-cell sequencing is a powerful technology for investigating ITH by identifying genomic alterations and distinct transcriptomic states in single tumor cells [11–19]. For example, in clinical samples of glioblastoma, single-cell RNA sequencing showed that individual tumor cells vary in terms of their degree of stemness-related gene expression from extremely stem-like to differentiated states [13]. Additionally, the existence of cancer stem cells that continuously differentiate into astrocyte- and oligodendrocyte-like cells has been demonstrated in oligodendrogliomas by single-cell RNA sequencing [14]. Single-cell DNA sequencing has also been applied to breast cancer samples to evaluate ITH originating in genomic DNA, leading to the suggestion of stepwise/sweepstake or gradual evolution of cancer cells from single-nucleotide variation (SNV) data [11, 12, 20]. However, these types of ITH and their respective evolutionary mechanisms are based on snapshot data at one time point. Furthermore, either RNA or DNA was solely examined. It is necessary to address both RNA and DNA over time for the full elucidation of tumor evolutionary dynamics associated with ITH.

Mouse models are convenient for controlled serial time sampling to effectively examine changes in genomic and transcriptomic states over time, which is practically unrealistic with human samples. In a breast tumor xenograft mouse model, single-cell DNA sequencing of serially passaged samples identified tumor cell subpopulations and suggested that tumor cells in the same initial state followed the same evolutionary trajectory [21]. In the present study, we employed a modified version of the mouse colorectal cancer model that we previously established [22] and sequenced both single-cell DNA and RNA. In this model, a normal intestinal cell is grown into organoids that model the structure of intestinal crypts. After RNAi-medicated downregulation of a tumor suppressor gene *APC*, the transduced organoids are serially transplanted into nude mice to allow exposure to in vivo microenvironments. In this way, the model can mimic the development of colorectal cancer in which a normal intestinal cell subjected to *APC* impairment initiates uncontrolled cell proliferation that, together with interactions with the intestinal microenvironment, ultimately leads to the development of cancer with ITH. We thus investigated how ITH based on the exome and transcriptome changes over time at the single-cell level.

## Results

### Colorectal cancer mouse model

**Fig. 1:**
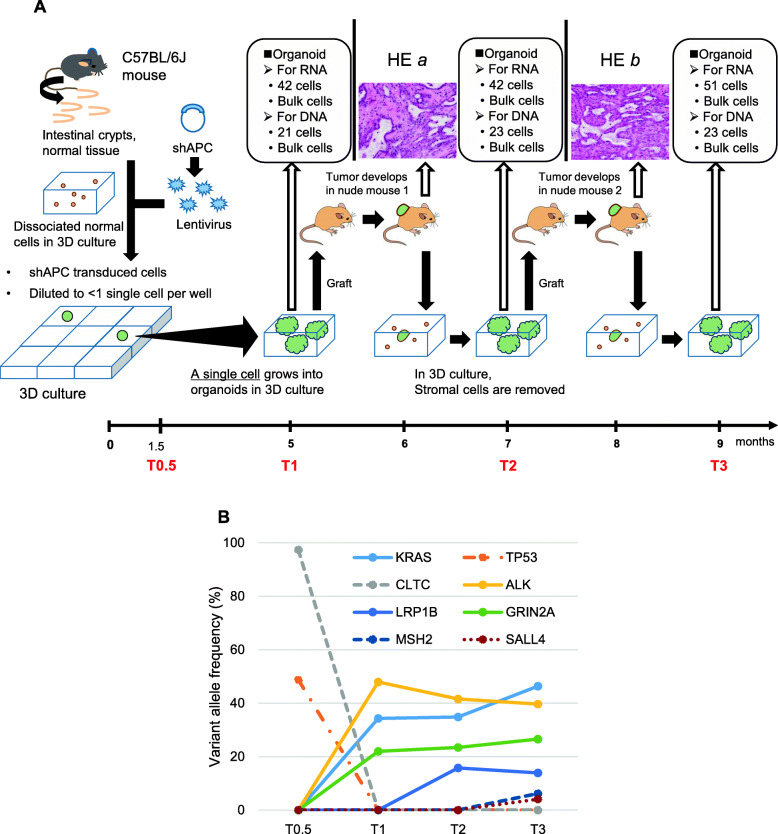
The mouse model. **A** The experimental procedure and HE staining of subcutaneously transplanted tumors. *One single cell* was 3D-cultured in a 96-well plate to grow organoids (see “Methods” for details). Single cell-derived organoids were taken to separate single cells, and RNA and DNA were separately extracted from the different single cells of multiple organoids and then sequenced. The numbers of cells for RNA and DNA sequencing in boxes are those obtained after quality control of data. The culture experiment from intestinal crypts to T3 organoids was performed once. **B** Variant allele frequencies of mutations found in the significantly mutated genes of colorectal cancer by bulk-cell DNA sequencing. See Additional file 1: Figure S1 for the annotations of the mutations

The colorectal cancer mouse model was established by knocking down *APC* expression in normal epithelial cells taken from mouse intestinal crypts using short hairpin RNA (shAPC; Fig. 1A) [22]. In the previous system, we used bulk cells from a tissue for culture; however, in this study, we cultured organoids from *one single cell* so that heterogeneity observed in these cultures could not be confused with heterogeneity originating from the knockdown efficiency or intestinal crypts [23]. We grew organoids for a period of 5 months so that the initial single cell with only artificial *APC* intervention could naturally obtain mutations to transform into tumor cells. The culture experiment was performed once; see “Methods” for the experimental details.

Cultured cells were subcutaneously transplanted into a nude mouse. One month after transplantation, the mouse was sacrificed, and the tumor was harvested; half of the tumor tissue was re-cultured in our three-dimensional (3D) culture system for 1 month for the removal of stromal cells. Using half-samples preserved the same genetic lineage over time. The process was repeated once more. Cells were sampled immediately before the first transplantation at time point T1 and at two time points T2 and T3 following the first and second transplantations, respectively (Fig. 1A). We sequenced single-cell RNA and DNA separately taken from the different single cells of multiple organoids, which descended from one single cell. We also obtained a DNA sample before T1, at T0.5, 1.5 months after culture initiation (Fig. 1A).

Hematoxylin-eosin (HE) staining revealed that subcutaneously transplanted organoids formed tumors consisting of both glandular and non-glandular structures (HE *a* and *b* in Fig. 1A). Glandular components in HE *a* were mainly lined with single-layered epithelia, while those in HE *b* were characterized by increased multi-layered regions, loss of cellular polarity, and nuclear enlargement. Non-glandular components had a stromal/medullary structure consisting of spindle-shaped or round to polygonal cells, were characteristically gelatinous/fibrous, and had an abundance of fibrous stroma.

The *APC* expression was decreased in the *APC* knockdown samples (Additional file 1: Figure S1). Out of the 31 significantly mutated genes (excluding *TTN*) defined by The Cancer Genome Atlas (TCGA) colorectal cancer study [24], we found two mutations in *KRAS* and *TP53* by bulk-cell DNA sequencing in our model (Fig. 1B), though the *KRAS* mutation was located outside of, but close (9 bps) to, an exon and the position was evolutionary conserved as much as exons (Additional file 1: Figure S1). The *KRAS* mutation occupied only a small fraction (2.5%) of the population at T0.5 but increased to 46.4% at T3. Additionally, we found nonsynonymous mutations in six, *CLTC*, *LRP1B*, *ALK*, *GRIN2A*, *MSH2*, and *SALL4* out of the cancer-related genes in COSMIC Gene Census [25] (Fig. 1B). It seems that a major clone with the *TP53* mutation might have existed alongside minor clones without this mutation at T0.5 (considering a VAF of 48.7% at T0.5, 1–2% minor clones are possible). A minor clone with the *ALK* and *KRAS* mutations replaced the major clone between T0.5 and T1, during which the number of SNVs drastically increased, as we will show later.

### Single-cell transcriptome analysis

We checked various indices of single-cell transcriptome data to filter 42, 42, and 51 cells out of the 50 T1, 43 T2, and 52 T3 cells, respectively (Additional file 1: Figure S2). The median (± interquartile range) number of mapped reads, mapping rate, and number of expressed genes across selected cells were 6.2 × 10^6^ (± 2.0 × 10^6^), 61.9% (± 5.39%), and 3814 (± 889.5), respectively. There was a strong correlation between gene expression levels in the bulk sequencing data and average expression levels across single cells (Additional file 1: Figure S2; *R*^2^ = 0.9). The expression levels of housekeeping genes (*GAPDH* and *mtATP6*) [26] were well reproduced across T1, T2, and T3 (Additional file 1: Figure S2). We performed a bootstrap approach where we re-sampled sequence reads and re-aligned them to obtain bootstrapped expression data, and estimated that the replicate variability of the expression levels (relative errors of log_2_[TPM + 1] values) was about 1% on average for a single cell (Additional file 1: Figure S2).

**Fig. 2:**
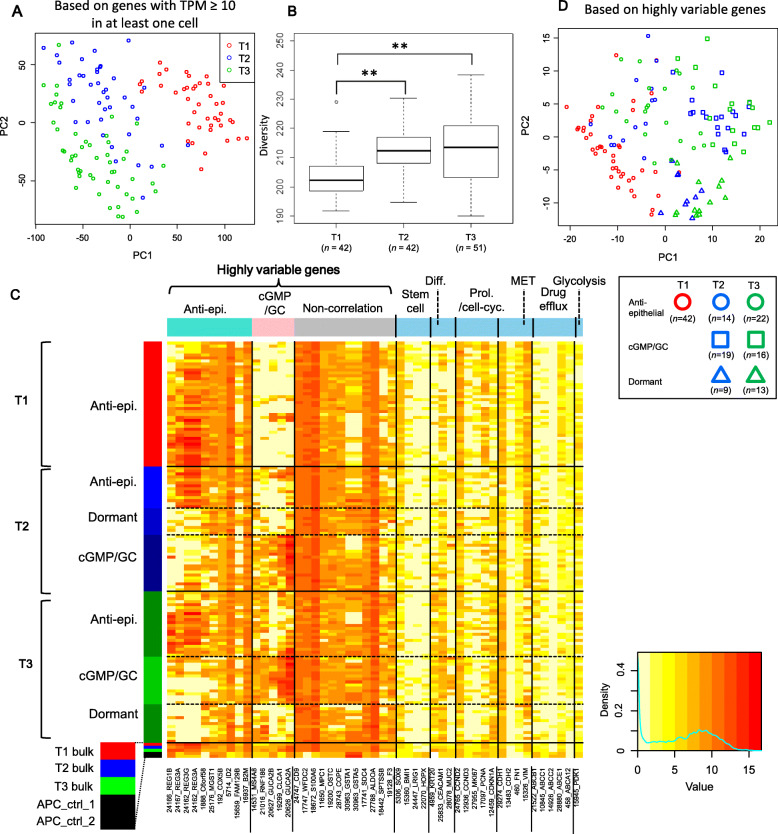
Transcriptome analysis. **A** PCA plot of single cells based on expression levels (genes with TPM ≥ 10 in at least one cell). T1, at the time of 3D culturing; T2 and T3, after the first and second transplantations, respectively. *n* = 42, 42, and 51 cells for T1, T2, and T3, respectively. **B** Boxplots of Euclidean distance from the centroid in the PCA space (using full dimensions). **: *P* < 0.01 (two-sided Wilcoxon rank sum test). **C** Heatmap of gene expression levels (in TPM). The rows represent single cells or bulk-cell samples (in the bottom), and the columns represent highly variable genes and several types of marker genes. The cell and gene groups were determined as shown in Additional file 1: Figure S4. The red, blue, and green codes in the rows correspond to T1, T2, and T3. “Diff.” and “Prol./cell-cyc.” represents differentiation and proliferation/cell cycle. “APC_ctrl” indicates control samples that were cultured in our 3D culture system and derived from normal cells without *APC* knockdown. **D** PCA plot of cells grouped based on expression levels of highly variable genes

A principal component analysis (PCA) plot of cells based on expression levels revealed increased diversity from T1 to T2 (Fig. 2A). This was quantitatively confirmed by the diversity index (distance from the centroid in the PCA space) (Fig. 2B). In the plot, T2 and T3 cells partly overlapped but were separate from T1 cells. We identified genes whose expression levels varied greatly across cells at each time point; that is, these genes had high corrected coefficient of variation (c*CV*) values (Additional file 1: Figure S3), and were thus referred to as highly variable genes. There were 8, 14, and 16 highly variable genes at T1, T2, and T3, respectively, reflecting an increase in variability from T1 to T2.

A cluster analysis of highly variable genes identified three gene groups (Additional file 1: Figure S4); expression levels were correlated within two of the groups, but not within the third group. Gene set enrichment analysis showed that one of the correlated groups was associated with negative regulation of keratinocyte differentiation (referred to as anti-epithelial genes) (*P* = 3.80 × 10^− 3^), whereas the other was associated with positive regulation of cGMP and guanylate cyclase (GC) activity (referred to as cGMP/GC genes) (*P* = 1.30 × 10^− 3^), which are known to be associated with negative regulation of β-catenin signaling and matrix metalloproteinase activity in colorectal cancer [27, 28].

A heatmap generated from the cluster analysis revealed that T1 cells were relatively homogenous and formed one group that highly expressed anti-epithelial genes but showed negligible expression of cGMP/GC genes (Fig. 2C). This group was therefore termed anti-epithelial. In addition to an anti-epithelial cell group, two new groups appeared at T2: one showing the opposite pattern, repression of anti-epithelial and activation of cGMP/GC gene expression, referred to as the cGMP/GC cell group; the other showed repression of both anti-epithelial and cGMP/GC genes, referred to as the dormant group for the marker analysis described below. Notably, as shown in the heatmap, bulk-cell sequencing analysis alone could not have identified these cell groups, where their distinct expression patterns were averaged in bulk-cell expression levels (labeled as T1, T2, and T3 bulk in Fig. 2C). Using a PCA plot based on highly variable gene expression, we confirmed that T1 cells were relatively homogeneous and T2 cells showed similar grouping to T3 cells (Fig. 2D).

### Marker gene expression

We examined the expression of several types of marker genes. *MKI67* and *PCNA* were used as positive markers, and *CDKN1A* was used as a negative marker for cell proliferation in colorectal cancer [29]. *CCND2* and *CCND3* were used as positive markers for cell cycle in colorectal cancer [30]. E-cadherin (*CDH1*) is an epithelial marker, and N-cadherin (*CDH2*), vimentin (*VIM*), and fibronectin (*FN1*) serve as typical mesenchymal markers [31]. *LGR5*, *ASCL2*, *OLFM4*, *MSI1*, and *SOX9* are crypt base stem cell markers; *HOPX*, *BMI1*, and *LRIG1* are the + 4 (position from the crypt base) stem cell markers; *AQP8*, *CAR1*, *CEACAM1*, *KRT20*, and *SLC26A3* are differentiation makers for absorption cells; and *MUC2* and *SPINK1* are differentiation markers for secretion cells [32].

**Fig. 3:**
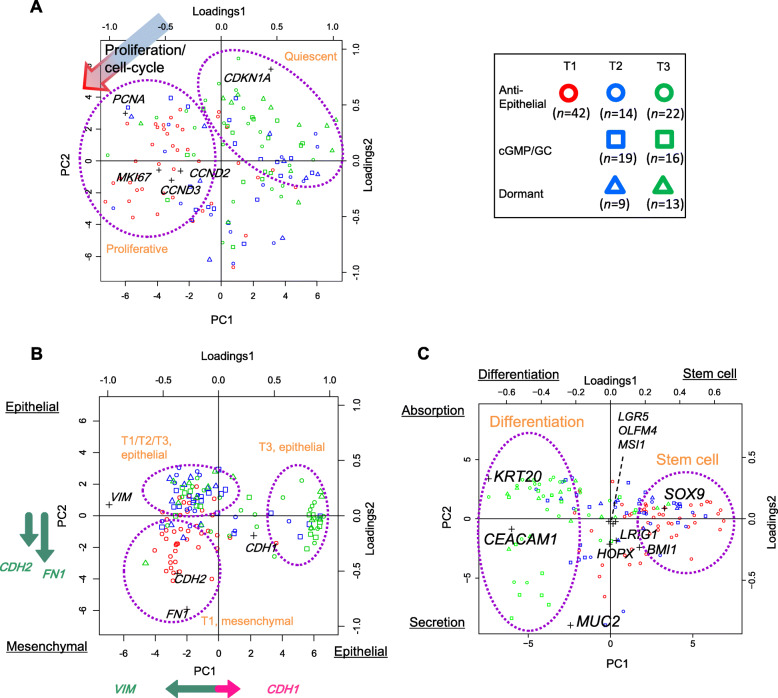
PCA and overlaid loading plots based on expression levels of markers. **A** About the proliferation/cell cycle. The arrow indicates the direction from negative to positive markers in the loading plot; cells positioned in that direction in the PCA plot had higher expression levels of positive marker genes. **B** About the epithelial and mesenchymal. The arrows along the *x* and *y* axes represent projected loadings in the loading analysis, where cells positioned in that direction in the PCA plot had higher marker gene expression levels. **C** About stem cell and differentiation

We first looked at proliferation/cell cycle markers (Additional file 1: Figure S5) and performed PCA to summarize the multiple expression levels (Fig. 3A). Remarkably, most cells in the anti-epithelial group at T1 expressed high levels of proliferation- and cell cycle-related genes according to the PCA loading plot (left circle, drawn by hand). In contrast, nearly all cells in the dormant group at T3 showed a downregulation of the marker genes (right circle; so the cell group was termed dormant). At T2, about half of the cells showed a downregulation of the proliferation/cell-cycle genes.

We next examined epithelial and mesenchymal markers (Additional file 1: Figure S5). A PCA plot of the markers showed that expression of mesenchymal cell-related genes decreased with time (T2 and T3, moving from the lower left to the upper left to the middle right circles), with cells forming two groups (Fig. 3B): one (upper left circle) overlapping with some T1 anti-epithelial cells with decreased mesenchymal N-cadherin (*CDH2*) and fibronectin (*FN1*) levels; the other (middle right circle) group was composed only of T3 cells with decreased mesenchymal vimentin (*VIM*) and increased epithelial E-cadherin (*CDH1*) levels. These results suggest a similarity between the processes occurring from T1 to T2 and mesenchymal-epithelial transition (MET).

Stem cell and differentiation markers showed that over time cells generally expressed more differentiation than stem cell markers (Fig. 3C, from the right to left circles; Additional file 1: Figure S5), though a remarkable variation across individual cells was also observed. Fractions of cells with the expressions of stem cell markers decreased with time. Among the markers for crypt base stem cells, *SOX9* appeared to be the most influential; *LGR5*, *OLFM4*, and *MSI1* were not substantially expressed. It seems that with time, cells differentiated into those expressing a marker for absorption cells (*KRT20*; cells in the upper left part) and those for secretion cells (*MUC2*; cells in the lower left part) in the digestive tract.

There was no remarkable change in the expression of drug efflux genes [33, 34] at any time point (Fig. 2C), although *ABCB1* expression was slightly lower in the T3 dormant group (Additional file 1: Figure S5) and *ABCE1* was downregulated at T2 and T3. There was variable expression of glycolysis-related gene *PDK1* [34] across all cells, irrespective of groups (Fig. 2C; Additional file 1: Figure S5).

### Single-cell exome analysis

Based on several indices from single-cell exome sequencing (Additional file 1: Figure S6), we selected 21, 23, and 23 cells out of the 23 T1, 24 T2, and 24 T3 cells for analysis. On average (expressed as the median [± inter quartile range] across selected cells), the number of mapped reads was 1.2 × 10^8^ (± 2.2 × 10^7^), mapping rate was 76.6% (± 4.9%), coverage with > 0 depth regions was 76.9% (± 34.2%), average depth was 43 (± 34.5), Gini coefficient was 0.85 (± 0.15), allelic drop-out (ADO) rate was 47.0 (± 36.1), and number of called SNVs was 462 (± 313.5). We compared the fractions of single cells with SNVs to the variant allele frequencies (VAFs) of the bulk-cell sequencing; in theory, the single-cell fractions should be equal to half of the VAFs. We confirmed a good concordance between these variables, although the cell fractions were slightly lower than those expected from bulk VAFs (Additional file 1: Figure S6).

We estimated the false-positive rate of SNVs called in single-cell sequencing, based on normal intestinal tract tissue samples from two mice and four single cells obtained from one of these samples. We first counted the number of SNV sites that differed between two individual mice of the pure C57BL/6J strain. For normal intestinal tract samples obtained from the two mice, we called SNVs in bulk-cell sequencing data using each of the two samples as the foreground data and the other as the background: the numbers were 1.0 and 4.5 × 10^− 7^ per chromosomal position for the two sample pairs, respectively. When we called SNVs in half-split sequencing data used as the fore- and background data for the same sample, the number of SNVs per position was 0 and 0.4 × 10^− 7^ for the two samples, respectively. Taken together, the false-positive rate in bulk-cell sequencing was estimated as 1.0–4.9 ([1.0 + 0.0]–[4.5 + 0.4]) × 10^− 7^. Next, because we called SNVs in single cells only at SNV sites called in bulk-cell sequencing data, the false-positive rate in single cells was not more than that in bulk-cell sequencing. Since 10–23% of chromosomal positions were called by our loose criteria for sequencing data from four single cells obtained from normal intestinal tract tissue, the false-positive rate per chromosomal position in single-cell sequencing was estimated as 0.1–1.1 × 10^− 7^.

**Fig. 4:**
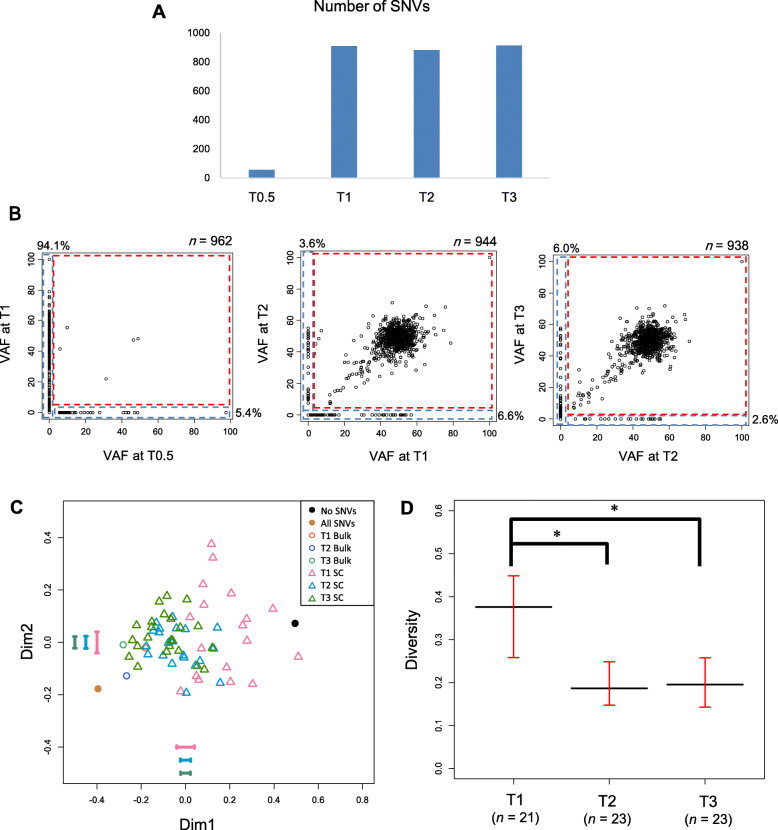
Exome analysis. **A** Number of SNVs called in bulk-cell sequencing. **B** Comparison of VAFs of SNVs called in bulk-cell sequencing at successive time points. One point indicates one SNV; *n* represents the number of points. **C** MDS plot based on single-cell exome sequencing. “No SNVs” and “All SNVs” represent sequences with no SNVs and with SNVs at all sites, respectively, which were artificially generated as a reference. Error bars represent the standard deviation for each dimension calculated with a bootstrapping approach that took into account ADO rates. *n* = 21, 23, and 23 cells for T1 SC, T2 SC, and T3 SC, respectively. **D** Median Euclidean distance from the centroid over cells in the MDS space. The black and red bars represent the observed value and 95% confidence interval calculated with the bootstrapping approach. *: *P* < 0.05 (bootstrapping test). *n* represents the number of cells

We first examined the bulk-cell sequence data. The T0.5 tissue had much fewer SNVs than the later stages (Fig. 4A), which suggests that DNA heterogeneity only weakly appeared soon (1.5 months) after culture initiation. The numbers of SNVs increased markedly from T0.5 to T1, a 5-month period (Fig. 4A). Although these numbers decreased slightly at T2 before recovering at T3, they were all mostly saturated at T1, T2, and T3. Thus, new SNVs were largely generated from T0.5 to T1, and most of these SNVs remained in the genome after T1 at the bulk-cell level (Fig. 4B).

We then used single-cell sequencing data to draw a multidimensional scaling (MDS) plot based on single-cell SNVs at polymorphic SNV sites (defined as SNVs with 10–35% bulk VAFs) (Fig. 4C). T1 cells showed the greatest genetic diversity, whereas T2 and T3 cells showed less diversity. This decrease in diversity was confirmed by a statistical significance of the diversity index (average distance from the centroid), where the bias due to ADO rates was taken into account by a bootstrapping test (Fig. 4D). Interestingly, this diversity tendency was the complete opposite of the transcriptomic pattern (Fig. 2A, B).

### Association with human cancer

We examined whether the identified cell groups featured by expression of genes had an association with malignancy represented by metastasis. We searched the TCGA dataset [24, 35] for human colorectal cancer samples with expression patterns similar to those of the genes characterizing the mouse cell groups. For example, if a TCGA sample is *predominantly* composed of a tumor cell group with a similar expression pattern to that of the genes characterizing the anti-epithelial mouse cell group, the expression pattern of the TCGA sample should be close to that of the anti-epithelial mouse cell group, despite the fact that the TCGA data are derived from bulk-cell sequencing.

**Fig. 5:**
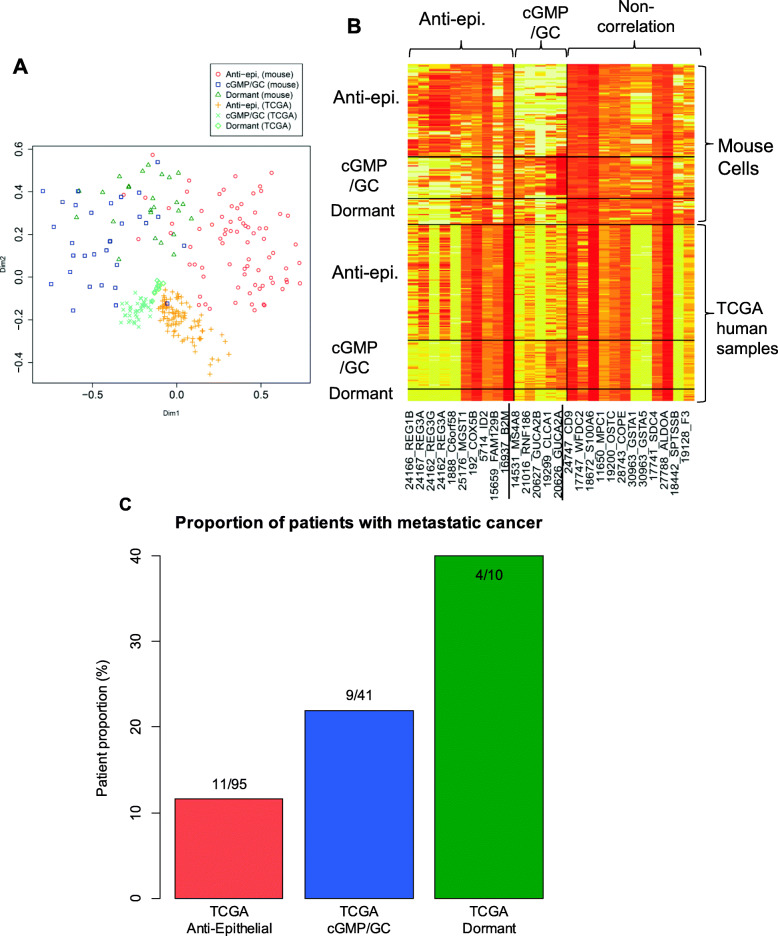
Analysis of TCGA human samples with gene expression patterns similar to mouse cell groups. **A** MDS plot of mouse single-cell samples and such TCGA samples on the basis of a similarity of gene expression patterns. *n* = 42, 42, and 51 cells for the anti-epithelial, cGMP/GC, and dormant groups, respectively; *n* = 94, 42, and 13 TCGA samples for the anti-epithelial, cGMP/GC, and dormant types, respectively. **B** Heatmap of the samples. Genes are highly variable genes shown in Fig. 2C. The number of cells and cases are the same as in **A**. **C** The fraction of patients with metastatic tumor in TCGA samples with expression patterns similar to mouse cell groups

In MDS analysis (Fig. 5A), among the total 244 TCGA samples, we identified 149 samples with similar expression patterns to any of the mouse cell groups: 94 (38.5%), 42 (17.2%), and 13 (5.3%) TCGA samples were respectively close to the anti-epithelial, cGMP/GC, and dormant mouse cell groups. TCGA anti-epithelial samples showed enhanced *REG* and repressed cGMP/GC gene expression; TCGA cGMP/GC samples showed the opposite pattern; and TCGA dormant samples had both repressed *REG* and GC-related gene expression (Fig. 5B). Next, we examined an association between these TCGA samples and metastasis. TCGA cGMP/GC and TCGA dormant samples tended to be more closely associated with metastasis than those with patterns similar to the anti-epithelial group (two-sided Fisher’s exact test *P* = 0.04; Fig. 5C).

In addition, we examined whether our mouse cancer model corresponds to the hypermutation type of human colorectal cancer, using our mouse bulk sequencing data and TCGA human colorectal cancer data. SNV density in the mouse model was closer to the hypermutation type of human colorectal cancer (Additional file 1: Figure S7). The expression of *MLH1*, the dysregulation of which causes hypermutation, was repressed with the levels decreasing over time (from T1 to T3) (Additional file 1: Figure S7). The average copy number across the mouse genome was closer to the hypermutation type, indicating low chromosomal instability (Additional file 1: Figure S7). Taken together, these results suggest that the mouse model was closer to the hypermutation type (albeit not extremely hyper) of human cancer.

We further analyzed corresponding histological types and microsatellite instabilities in a machine learning approach (random forest) using a histological type or microsatellite instability as the objective variable and omics (SNV/indel/RNA) data as explanatory variables. Of the three histological types, including colon and rectal mucinous adenocarcinoma, our mouse model was closest to human colon adenocarcinoma, and was closer to the MSI-high than MSI-low and microsatellite-stable types (Additional file 1: Figure S8). Thus, our mouse model represented the MSI-high hypermutation (although, not extremely hyper) type of human colon adenocarcinoma.

## Discussion

Our mouse model was close to human colon adenocarcinoma of the MSI-high hypermutation type. In this model, once cancer cells accumulate a sufficient number of genetic alterations (SNVs/indels), they may be able to adapt to drastic environmental changes, such as the shift from a 3D culture to a live mouse, by only altering their transcriptional profiles without further genetic changes. Such transcriptional adaptation may cause the generation of new subpopulations, leading to increased transcriptional heterogeneity. Meanwhile, genetic heterogeneity decreased, possibly as a result of microscale natural selection that occurred during the environmental transition. Though expected, it is nonetheless surprising to see that this diversity was indeed generated from *one single cell*. One caveat is that the outcome of the evolutionary path could be unique to the given experiment, because the experiment from a single cell to organoids after allotransplant at T3 was performed only once.

T1 cells had the saturated number of genetic mutations, expressing active cell cycle, mesenchymal, and stem cell markers. Thus, the cells are considered as those at a late tumor stage when they move out from the niches or microenvironment of intestinal crypts [36]. Moreover, the emergence of the dormant and cGMP/GC groups at T2 and T3 was associated with metastasis in the analysis using TCGA human samples. Therefore, our observation that cells lose their mesenchymal gene expressions and acquire epithelial-like characteristics after subcutaneous transplantation may be analogized to MET during metastasis, though this implication should be tested by further investigations of clinical samples including single-cell sequencing of TCGA samples.

Considering the decreased DNA ITH and dominance (90%, 883 / total 987 sites) of fixed SNVs over polymorphic SNVs, tumor cells were close to be genetically monoclonal based on DNA-seq, which is in contrast to the high ITH and presence of subpopulations detected using RNA-seq. Using SCmut [37], a tool specialized for calling SNVs in single-cell RNA-seq, we attempted to find a link between the small genetic differences and RNA subpopulations by comparing single-cell DNA SNVs and single-cell RNA SNVs. However, we did not find a clear association. Simultaneous single-cell sequencing of both DNA and RNA from the same cells ([38, 39]) may be required for further clarification.

Classically, cells that generate a tumor by subcutaneous transplantation are called tumor-initiating cells or cancer stem cells (CSCs) [34]. In this classical model, it is expected that differentiated cells die while CSCs can survive at the start of subcutaneous transplantation and 3D culture; then, CSCs re-generate differentiated cells. We initially expected that fractions of cells expressing stem cell markers increased over the serial transplantation, because we simply thought that it is CSCs, not differentiated cells, that can survive at transplantation. However, our observation was the opposite. CSCs that efficiently generate differentiated cells may be more adaptive for the merit of obtaining mutual benefits between different types of cells. Alternatively, contrary to the classical expectations, CSCs may not necessarily express high levels of stem cell markers: the dormant and cGMP/GC cells with low expression levels of stem cell markers may be also CSCs or tumor-initiating cells that survived at the transplantation and 3D culturing.

Nguyen et al. [40] used DNA barcoding for breast cancer xenografts to find several patterns of clone size dynamics across clones and samples at serial transplants; however, DNA changes in the genomes after the insertion of barcodes were not taken into account. Eirew et al. [21] sequenced the genomes of breast cancer patient-derived xenografts to reveal that the same starting tumor cell population could result in similar dynamic patterns of clone size growth at serial passages. However, neither of these studies included transcriptome analysis. Roerink et al. [41] performed multi-regional sequencing for single cell-derived organoids of colorectal cancers at a single time point to reveal that even genetically close cancer cells exhibited marked differences in responses to anticancer drugs as measured by the IC_50_ value, although the transcriptome dynamics related to these response differences were not analyzed. We sequenced both the DNA and RNA of single-cell-ancestral allografts of colorectal cancer at serial passages and revealed that genetically similar cancer cells with sufficient DNA changes diverged into two new transcriptional subpopulations to respond to drastic environmental changes such as serial passages.

It is important to confirm data quality in single-cell sequencing. For RNA, we performed a quality control (QC) check and filtered out cells such as those with too many or few expressed genes and confirmed a good correlation in the expression levels between bulk- and single-cell sequencing (Additional file 1: Figure S2). We further confirmed high concordance in the expression levels of housekeeping genes between bulk- and single-cell sequencing across all time points, and the estimated replicate variabilities (bootstrapped relative errors) of the expression levels were clearly smaller than the inter-subgroup differences (Additional file 1: Figure S2). For DNA, we used the multiple displacement amplification method for whole-genome amplification because of the reduced amplification bias compared to PCR-based amplification [42]. We also performed a QC check and cell filtering (Additional file 1: Figure S6) in the same manner as performed for the RNA data. Moreover, SNVs were called in single cells only when the same SNVs were called in bulk cells and, as expected, the estimated false-positive rate was quite small. Nevertheless, we recognize that a high ADO rate may restrict available analyses. For an analysis that could have been affected by ADO, we adjusted for the ADO by a bootstrapping method (see error bars in Fig. 4C, D). Overall, these processes confirmed the quality of our single-cell sequencing data.

Recently, more fine-scale single-cell sequencing technology, such as 10X/Drop-Seq, has emerged for RNA-seq, enabling researchers to capture tens of thousands of cells. Although the number of cells we addressed was relatively small compared to that technology, we believe that we successfully captured a major part of the heterogeneity constructed by cell clones, constituting as small as ~ 2% (an inverse number of 42, 42, and 51 cells at T1, T2, and T3) of the tumor cell population. Nevertheless, 10X/Drop-Seq will be needed to investigate rarer cells.

## Conclusions

We demonstrated that time-series ITH analysis by single-cell DNA and RNA sequencing for a mouse model is able to deepen our understanding of the evolutional processes of cancer cells and raise issues on CSCs from the genomic and transcriptomic viewpoints. The birth of transcriptional subpopulations of cells may be a key for adaptation to drastic micro-environmental changes when cancer cells have sufficient genetic alterations at later tumor stages. It will be crucial to examine how such genetic changes accumulate in the earlier stages of tumorigenesis and how transcriptional subpopulations develop to increase malignancy in the further later stages of tumor progression.

## Methods

### Organoid culture of small intestinal cells and lentiviral transduction

C57BL/6J mice and BALB/cAnu/nu immune-deficient nude mice were purchased from CLEA Japan (Tokyo, Japan). The small intestine was harvested from wild-type male C57BL/6J mice at 3–5 weeks of age (Additional file 1: Figure S9A). Crypts were purified and dissociated into single cells, which were then put into serum-free Matrigel-based organoid culture as previously described [22, 43]. Five days later in the first passage, organoids were lentivirally transduced with shRNA against APC, where the efficiency of introducing the shRNA was around 90% [22, 43]. To select for APC-repressed cells, transduced organoids were thereafter maintained in culture medium lacking R-spondin 1, which activates Wnt pathway and is thereby indispensable for propagation of normal intestinal cells. To obtain a single-cell clone, shAPC-transduced organoids were dissociated and plated at the concentration of 0.5 cell/well in a 96-well plate. Immediately after plating, 50 wells containing only a single cell were identified under microscope; the remaining 46 wells were occupied by no cells or 2 or more cells. Then, we selected 24 out of the 50 single-celled wells and placed the organoids from each well into a separate well of a 24-well plate. Fresh culture media at the passage were necessary for removing wastes and multiplying organoids. We repeated these procedures for 5 wells selected from among the 24 wells, and finally specified 1 well for later use as organoids originating from a single cell.

We then grew organoids from a single cell in an individual well, transferring the fastest growing organoids from 1 to 2 to 4 to 8 wells as they multiplied (Additional file 1: Figure S9B). On the way to the 8-well stage, as is usually done in this type of culture, we collected organoids into a single tube at every passage, resulting in the intermixing of organoids across different wells. At the 8-well stage, we collected organoids from 4 out of the 8 wells into a single tube for storage and also sampled the DNA for sequencing, which was used as the T0.5 sample. Two of the remaining wells were used for re-culture, and two were used for the mouse injection test; the same procedure was used for T1, T2, and T3 (Additional file 1: Figure S9B). Organoids composed of 5 × 10^5^ cells were mixed with 200 μl of Matrigel and injected into one side of the dorsal skin of nude mice, while uninjected cells were maintained in 3D cultures for later use.

### Analysis of subcutaneous tumors in nude mice

Palpable tumors from the injection sites were harvested for histological examination or cell culture. Half of the subcutaneous tumors were formalin-fixed, paraffin-embedded, and sectioned at a thickness of 5 μm, followed by HE staining to assess histological features. The other half of the tumors were minced and digested to recover cells as described previously [22], then seeded onto solidified Matrigel to resume organoid culture.

### Single-cell transcriptome and exome sequencing

Cultured mouse organoids derived from a single cell were harvested and treated with Accumax (Innovative Cell Technologies, AM105) to generate a single-cell suspension. To capture cells and extract RNA or DNA from a single cell, the cell suspensions (4.4 × 10^5^ cells/ml) were loaded on a C1 Single Cell Auto Prep System (Fluidigm, C1) with medium-sized (10–17 μm) microfluidic chips for 96 cells. Approximately 1300 cells were applied to each chip. Captured cells were imaged on a BZ-9000 digital microscope (Keyence, BZ-9000) and a visual inspection was performed to determine whether a single cell was captured in each well of the chip. Capture efficiency for a single cell was determined as 71–82%.

For single-cell whole transcriptome (RNA) sequencing, captured cells were lysed on the chip using a C1 Single-Cell Auto Prep Reagent Kit for mRNA Seq (Fluidigm, 100-6201). Full-length cDNA fragments were transcribed and amplified from poly-A RNA in each single cell using the SMARTer Ultra Low RNA kit (Takara Bio, 634832). Tagmentation of cDNA was performed and sequencing libraries were prepared using the Nextera XT DNA sample preparation kit (Illumina, FC-131-1096) according to the manufacturer’s protocol. Up to 52 independent single-cell RNA-seq libraries were prepared for sequencing.

For single-cell DNA sequencing, genomic DNA was prepared from single cells using the C1 Single-cell Auto Prep Reagent Kit for DNA Seq (Fluidigm, 100-7357) and whole-genome amplification was achieved by multiple displacement amplification with Phi29 DNA polymerase and the Illustra GenomiPhi v.2 kit (GE Healthcare, 25660032). Amplified genomic DNA (70 ng) was used to generate exome sequence libraries using the Hyper Prep kit (Kapa Biosystems, KK8504), SureSelect Target Enrichment kit (Agilent Technologies, 931171), and SureSelect XT Mouse All Exon v.1 probe (Agilent Technologies, 5190-4642).

### Bulk-cell transcriptome and exome sequencing

Among the cells that were not used for single-cell capture with the C1 system, suspensions of about 200 cells were subjected to whole transcriptome (RNA) sequencing for bulk-cell RNA-seq (T1, T2, and T3 samples). The sequencing libraries were prepared using the same reagents as the single cell RNA-seq. As control bulk cells, normal intestinal crypt epithelial cells from two wild-type mice of the same strain were grown in the 3D culture system for 7 days, then harvested and lysed for total RNA preparation using the miRNAeasy Mini kit (Qiagen, 217004). RNA-seq libraries for control bulk RNA were generated using the SureSelect Strand Specific kit (Agilent Technologies, G9691B). Bulk DNA from over 1 × 10^5^ cells was obtained from the cell culture (T0.5 sample, 1.5 months after culture initiation) and the remaining cells in single-cell capture (T1, T2, and T3 samples) using the QIAamp DNA Mini kit (Qiagen, 51304), and 500 ng of DNA were used to construct exome sequencing libraries with the same reagents as the single cell DNA-seq.

### Sequencing

All the sequencing libraries were subjected to paired-end sequencing of 101-bp fragments on the HiSeq2500 DNA sequencer (Illumina, SY–401–2501).

### Transcripts per kilobase million (TPM) calculation for single and bulk cells

The procedure for calculating TPM is summarized in Additional file 1: Figure S10. Specifically, sequence reads were quality-filtered and trimmed using PrinSeq [44], and then used as input for quality-check by FastQC (https://www.bioinformatics.babraham.ac.uk/projects/fastqc/). We used the following parameters: --min_len 30 (removing reads ≤ 30 bases); --min_qual_mean 20 (average read quality ≤ 20); --trim_tail_right 5, --trim_tail_left 5 (trim bases if the 3′ and 5′ end poly A/Ts are ≥ 5 bases); and --trim_qual_right 20, --trim_qual_left 20 (trim 3′ or 5′ end for read quality ≤ 20). Paired-end reads were mapped to the University of California Santa Cruz mouse genome sequence (mm10) [45] using Bowtie2 [46] built in RSEM [47]. Expression levels (in TPM) were estimated by RSEM using the command rsem-calculate-expression with the parameters --estimate-rspd, --paired-end, --bowtie2, -p 30, and --ci-memory 10192. We removed eight T1 cell samples due to an excessive number of genes (≥ 5200) with TPM ≥ 10 (with reference to results in [48]) or with too few unique mapping reads (< 2.2 × 10^6^). We also removed two samples with unique mapping rates that were too low (< 20%) and discarded genes with low expression levels (≤ 10 TPM) across all cell samples, leaving 14,258 out of 32,732 genes for analysis.

### Estimation of replicate variabilities of gene expression levels

To estimate replicate variabilities of gene expression levels for a single cell, we first randomly selected three cells at each time point and then employed a bootstrapping approach where we re-sampled sequencing reads in the FASTQ file for each cell. We repeated this re-sampling three times for each cell to make three replicate sets of sequence reads. For each replicate set, we used the same method as for the observed data to obtain bootstrapped gene expression levels in log_2_ [TPM + 1]. We calculated the relative error of the expression level for each gene, setting the observed value in the denominator, and then averaged the relative errors across genes.

### Detection of highly variable genes

To detect genes with variable expression levels across cells, we defined highly variable genes according to the *CV*, corrected in the locally weighted scatterplot smoothing (LOWESS) method using the “lowess” function in R. To fit a single LOWESS curve across all ranges, we divided average expression level data into three ranges: < 4, 4–8.5, and > 8.5. c*CV* values were yielded by dividing *CV* values by the value of the upper variability band (± 1.96 times the standard deviation) of smoothed curve estimated using “loess.sd” in the “msir” package. Because of the large bias in original *CV* values against low average expression levels, only those with c*CV* values > 1.3 and high average expression levels (log_2_ [TPM + 1] ≥ 4) were defined as highly variable genes.

### PCA of RNA data

PCA was carried out for gene expression levels (log_2_ [TPM + 1]) without scaling.

### Hierarchal clustering, correlation plot, and heatmap analysis

For hierarchal clustering, we used the “hclust” function in the “stats” package of R software, where we calculated the Euclidean distance of expression levels (log_2_ [TPM + 1]) of all highly variable genes between cells and used the Ward method for agglomeration. We generated correlation plots of highly variable genes using the “corrplot” function in the R “corrplot” package, where we used the Ward method for agglomeration. We divided genes into three clusters based on these hierarchical clustering results using the “addrect = 3” option. A heatmap was generated using the “heatmap.2” function in the “ggplot2” package. In the heatmap, cells were arranged according to their order in the dendrogram described above and genes were arranged according to their order in the correlation plot of highly variable genes.

### Gene set enrichment analysis

DAVID [49] was used to identify gene ontologies (biological processes) in which genes of an identified group were enriched (*P* < 0.01).

### SNV detection for single and bulk cells

For bulk-cell data, we used a previously described method for SNV/indel calling [50] by cisCall with cell-line/frozen parameters [51], mapping reads to the mouse genome (mm9) [45] by BWA [52]. We filtered out PCR-duplicated reads as well as reads and bases with low mapping and base qualities. The remaining variants were further filtered statistically using Fisher’s exact test to compare fore- and background samples, which came from two different individuals of the same pure C57BL/6J strain. We verified the negligible effects of using a different individual for the background sample. A series of filters was used to remove suspicious variant calls, such as those related to misalignments. Variants for which allele frequencies were significantly greater than 1% in the binomial test were retained. The procedure is summarized in Additional file 1: Figure S10.

For single-cell sequencing data, we called SNVs only at SNV sites called in bulk-cell sequencing data. Specifically, we counted nucleotide bases with high qualities (mapQ ≥ 20, BaseQ ≥ 10) in single-cell sequencing data as well as in background data (same as those used in bulk-cell SNV calling) with the Samtools mpileup function [52, 53]. We then selected variants with the largest *χ*^*2*^ test statistic (with Yates’s correction) among all possible variants at each position to identify those that were most likely to differ between single-cell and background data. We required a variant count ≥ 2 and VAF ≥ 2% for such variants in single-cell data. We then selected variants that overlapped with SNV sites called in bulk-cell data.

### Detecting mutation in cancer-related genes

We investigated nonsynonymous mutations in cancer-related genes contained in Tier1 in COSMIC Cancer Gene Census v87 [25, 54].

### MDS of DNA data and the diversity index

We performed MDS from the cell × site matrix composed of zero and one, which respectively represent the absence and presence of SNVs (both synonymous and nonsynonymous SNVs) and NA, which represents an undetermined call due to shallow depth. We assigned zero to non-called sites as the true negative when those sites had depths ≥ 30 and assigned NA to non-called sites when the depth was < 30. We only used SNV sites where a variant was called in at least one cell and the VAFs at the same sites in bulk data ranged from 10 to 35% (polymorphic) for at least one time point. We removed cells and sites (two each) with too few or too many NAs, yielding 104 sites and 69 cells. Using this 0/1/NA matrix, we calculated the *p*-distance (proportion of different sites) used in molecular evolution without using NA, and then performed MDS.

The diversity index was calculated as the average Euclidian distance from the centroid over cells in the MDS space, where we used up to the sixth dimension because of a sudden drop in the eigenvalues over this dimension. To calculate the statistical significance of differences between cell groups, we used a bootstrapping approach in which we randomly re-sampled cells’ sequences from the 0/1/NA matrix of each cell group 10,000 times and performed the same MDS as in the observed data for each replicate. We then calculated the diversity index for each replicate to determine the 95% confidence interval and standard deviation for each cell group.

### Lorenz curve and Gini coefficients

A Lorenz curve was generated with read depth at each site using the “Lc” function in the “ineq” package of R software. To quantify uniformity, the Gini coefficient was calculated using the “Gini” function in the “ineq” package.

### ADO rate

The ADO rate was defined as the rate of homozygous sites in single-cell samples where the bulk sample was heterozygous (defined as sites where VAFs were 45–55%) at the same nucleotide site. We removed outlier cells with high ADO rates at each time point (one cell each with an ADO rate > 80% at T2 and T3).

### Average copy number

The average copy number, ACN, was calculated as follows:

1 $$ \mathrm{ACN}=2\times \left\{\left({2}^{\frac{\sum \left({\log}_2{R}_i\times {L}_i\right)}{\sum {L}_i}}\right)\times \left(\frac{\sum {L}_i}{GL}\right)+\left(1-\frac{\sum {L}_i}{GL}\right)\right\}, $$

where log_2_*R*_*i*_, *L*_*i*_, and *GL* represent the log-ratio of CNA segment *i*, length of CNA segment *i*, and genome length (50 Gb for mouse, 40 Gb for human), respectively. CNAs of mouse bulk data were detected as previously described [50]. Briefly, segments were called for the same fore- and background BAM files as those used in SNV with Exome CNV [55] and Varscan2 [56]. Overlapping segments called by both tools were used as CNA segments.

### Random forest

Random forest was used to generate the classifier for the histological type and MSI status of human cancer. We used gene expression levels, number of SNVs in each gene, total mutation (SNV/indel) number, and mutation density (total number of SNVs/indels divided by target region size) as explanatory variables. Using TCGA data [24, 35], we first filtered out unimportant explanatory variables using the two-sided Kruskal-Wallis test with *P* values of 5.00 × 10^−5^ and 1.00 × 10^− 9^ yielding 171 and 78 variables for histological type and MSI status, respectively. These were used to train a random forest classifier with the “randomForest” function in the “randomForest” package of R software, with the options ntree = 10000 (setting the number of trees to grow to 1000) and mtry = 5 (setting the number of variables randomly sampled to 5). Using the created classifier, the same explanatory variables for mouse data were used to classify each feature in the mouse model.

### MDS of mouse cell and TCGA samples

We first identified TCGA samples with gene expression patterns similar to the mouse single-cell groups. For that purpose, we calculated a normalized 1 − *r* distance as follows:

2 $$ {d}_{h,G}=\frac{1-{r}_{h,{m}^G}}{\mathrm{MADN}\left(1-{r}_{m_i^G,{m}^G}\right)}, $$

where *r*_*i,j*_ is a Pearson correlation coefficient between vectors *i* and *j* of expression levels in log across highly variable genes, *h* represents a human TCGA sample, *G* represents a mouse single-cell group, ${m}_i^G$ represents mouse single cell *i* in group *G*, *m*^*G*^ represents the centroid of ${m}_i^G$ that was calculated by the median, and MADN represents the median absolute deviation adjusted by a factor for asymptotically normal consistency. We calculated this distance from a TCGA sample to every mouse group and selected a TCGA sample for those whose minimum distance across the groups was less than 4.05 and the difference between the first and second minimum distances was larger than 0.31. For selected TCGA and all mouse single-cell samples, MDS was performed based on the distance of 1 − *r*.

### SCmut

We called SNVs in single-cell RNA-seq using SCmut [37], which is specifically designed to identify SNVs (heterozygous in a tumor sample but homozygous in the normal sample) in single-cell RNA-seq data with the aid of bulk-cell DNA-seq data. In their study [37], SCmut was applied to Illumina sequencing reads obtained using the Fluidigm C1 system, which is the same system that we used.

### Statistical analysis

All statistical analyses were performed using R (https://www.r-project.org/). Symbols “*” and “**” indicate *p* < 0.05 and 0.01, respectively, unless otherwise noted. The two-sided Wilcoxon rank sum test was used in the analysis shown in Fig. 2B. A bootstrapping test was used in the analysis shown in Fig. 4D. The details of this bootstrapping test are described above in the subsection “MDS of DNA data and the diversity index.” The number of samples (*n*) used in statistical analyses is indicated in each figure or figure legend as appropriate.

## Supplementary Information

**Additional file 1: Figure S1.** Aberrations in known cancer-related genes. **Figure S2.** Quality control check of single-cell transcriptome sequencing data. **Figure S3.** c*CV* and highly variable genes. **Figure S4.** Determination of gene and cell groups in single-cell RNA sequencing. **Figure S5.** Violin plots of the expression levels of the marker genes. **Figure S6.** Quality control check of single-cell exome sequencing data. **Figure S7.** Association with hypermutation type based on human cancer counterpart to our mouse model. **Figure S8.** Associations with histological type and microsatellite instability based on human cancer counterpart to our mouse model. **Figure S9.** Schematic representation of the culture experiment. **Figure S10.** Procedure for calculating expression levels and for calling SNVs in single-cell sequencing.

## Data Availability

The NGS sequence data generated and used in this study are available at https://figshare.com/articles/dataset/Single_Cell/14518056 [57]. Gene expression and clinical data of TCGA human colorectal cancers have been accessed from cBioPortal (https://cbioportal-datahub.s3.amazonaws.com/coadread_tcga_pub.tar.gz) [35]. Cancer Gene Census data have been accessed from the COSMIC website (version 87, follow instructions at https://cosmic-blog.sanger.ac.uk/downloading-previous-cosmic-releases/ or available from the corresponding author upon reasonable request and with permission of COSMIC, Wellcome Sanger Institute) [54]. The sequence data of the mouse reference genome and transcripts have been accessed from UCSC Genome Browser (UCSC Genome Browser assembly IDs: mm9, https://hgdownload.soe.ucsc.edu/goldenPath/mm9/chromosomes; and mm10, https://hgdownload.soe.ucsc.edu/goldenPath/mm10/bigZips/refMrna.fa.gz) [45].

## Abbreviations

ITH: Intra-tumor heterogeneity
PCA: Principal component analysis
ADO: Allelic drop-out
MDS: Multidimensional scaling
SNV: Single-nucleotide variation
TPM: Transcripts per kilobase million
LOWESS: Locally weighted scatterplot smoothing
VAFs: Variant allele frequencies
