# Supplementary material for: Single-cell DNA and RNA sequencing reveals the dynamics of intra-tumor heterogeneity in a colorectal cancer model
Source: BMC Biol. 2021 Sep 21;19:207. doi: 10.1186/s12915-021-01147-5 (PMC8456589; doi:10.1186/s12915-021-01147-5)
Supplement: Supplementary file 1 — Additional file 1: Figure S1. Aberrations in known cancer-related genes. Figure S2. Quality control check of single-cell transcriptome sequencing data. Figure S3. cCV and highly variable genes. Figure S4. Determination of gene and cell groups in single-cell RNA sequencing. Figure S5. Violin plots of the expression levels of the marker genes. Figure S6. Quality control check of single-cell exome sequencing data. Figure S7. Association with hypermutation type based on human cancer counterpart to our mouse model. Figure S8. Associations with histological type and microsatellite instability based on human cancer counterpart to our mouse model. Figure S9. Schematic representation of the culture experiment. Figure S10. Procedure for calculating expression levels and for calling SNVs in single-cell sequencing. [file 12915_2021_1147_MOESM1_ESM.pdf]

Fig. S1 (Part 1)

A

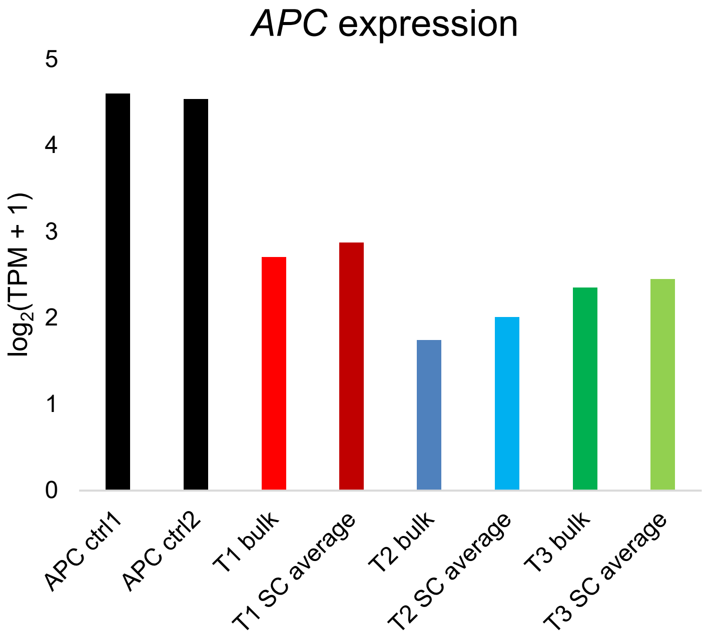

Fig. S1 Aberrations in known cancer-related genes. (A) Expression of the *APC* gene from bulk- and single-cell RNA sequencing. "APC_ctrl" indicates control samples that were cultured in our 3D culture system and derived from normal cells without *APC* knockdown at bulk-cell sequencing. "SC average" indicates the average across single cells at each time point. (B) Annotations of genes found in the significantly mutated genes of TCGA colorectal cancer samples and in COSMIC cancer-related genes by bulk-cell DNA sequencing. (C) The *KRAS* mutation in the mouse genome by the UCSC genome browser. The reversed U symbol in red indicates a mono-repeat of A. The arrow and line in gold indicate the position of the mutation.

Fig. S1 (Part 2)

B

HumanGene	Chr	Start	End	Mut_type	Ref	Alt	Notion	Reference
KRAS	6	145,169,253	145,169,253	indel	-	A	intronic	TCGA SMG
TP53	11	69,402,151	69,402,151	snv	A	T	nonsynonymous SNV	TCGA SMG and COSMIC
CLTC	11	86,520,656	86,520,656	snv	A	T	nonsynonymous SNV	COSMIC
ALK	17	72,952,883	72,952,883	snv	A	C	nonsynonymous SNV	COSMIC
LRP1B	2	40,724,718	40,724,718	snv	C	T	nonsynonymous SNV	COSMIC
GRIN2A	16	9,579,188	9,579,188	snv	T	C	nonsynonymous SNV	COSMIC
MSH2	17	88,079,144	88,079,144	snv	C	T	nonsynonymous SNV	COSMIC
SALL4	2	168,580,005	168,580,005	snv	C	T	nonsynonymous SNV	COSMIC

C

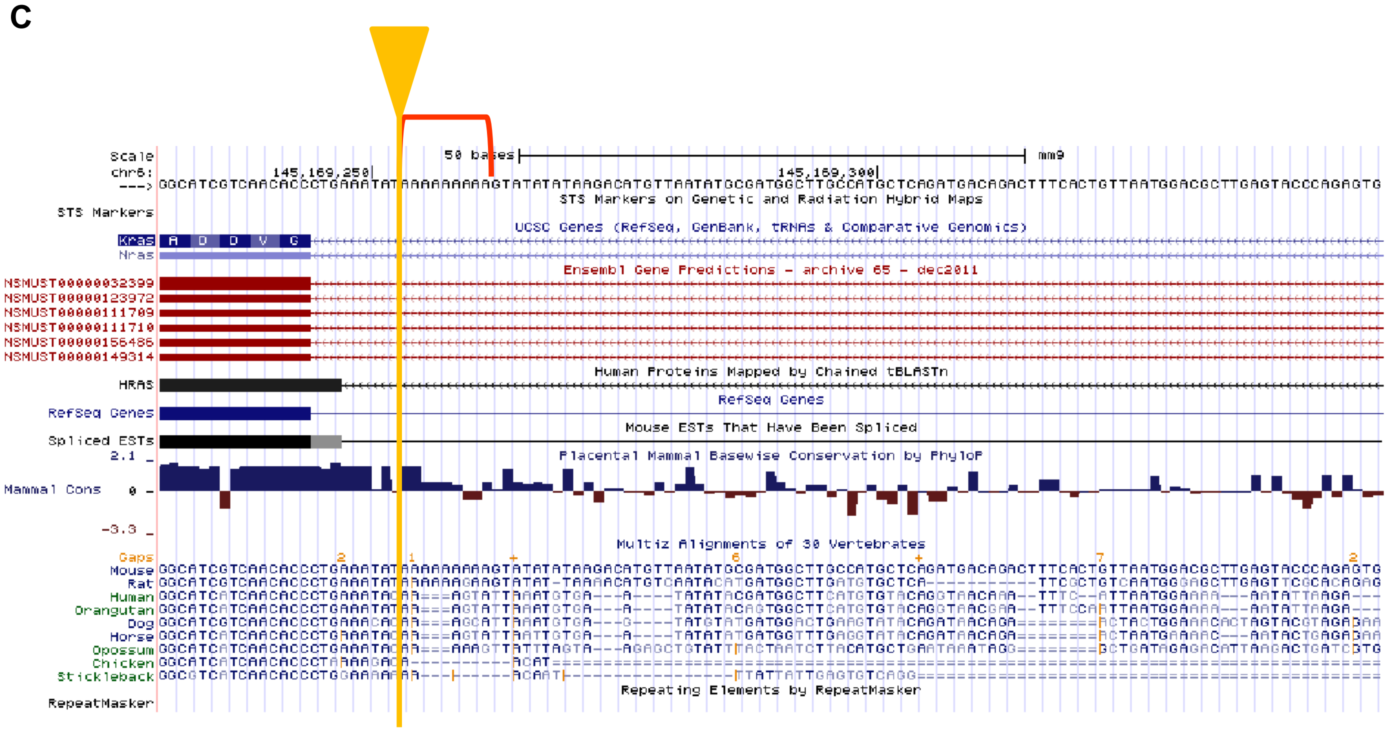

Fig. S2 (Part 1)

A

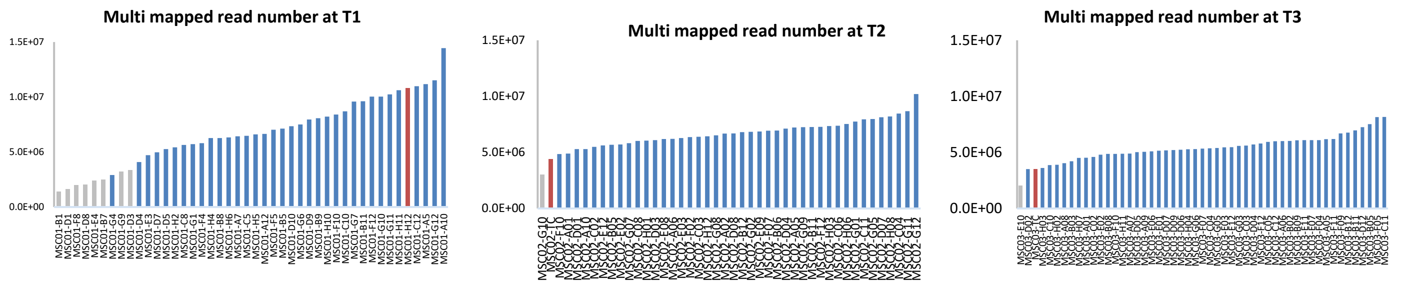

B

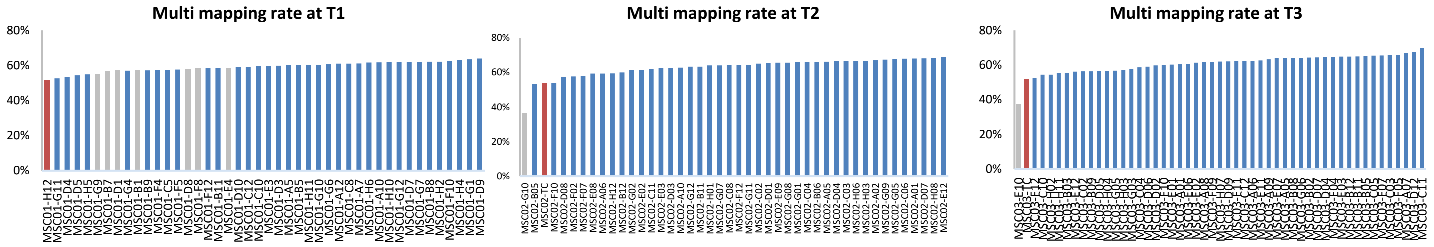

Fig. S2 Quality control check of single-cell transcriptome sequencing data. (A) Number of mapped reads, (B) mapping rate, and (C) number of expressed genes (TPM $\geq$ 10). We removed outliers (gray) based on the combination of the number of expressed genes ($\leq$5200) and number of mapped reads ($\leq 2.2 \times 10^6$), and the mapping rate ($\leq$20%). Blue and red bars represent single-cell samples that were ultimately used and bulk samples, respectively. (D) Scatter plot of gene expression levels from a bulk sample versus expression levels averaged across the single cells that were ultimately used. (E) Expression levels of housekeeping genes across T1, T2, and T3. (F) Estimated relative errors of expression levels. The estimation was made based on bootstrap re-sampling of sequence reads and re-mapping them to obtain bootstrapped expression levels in $\log_2$ (TPM + 1). We randomly selected three cells from each time point and for each cell we generated three bootstrap replicate sets of sequence reads. The labels such as "MSC01-B9" and "replicate 1" under the x-axis represent cell IDs and bootstrap replicate IDs, respectively. The relative errors were averaged across genes.

Fig. S2 (Part 2)

C

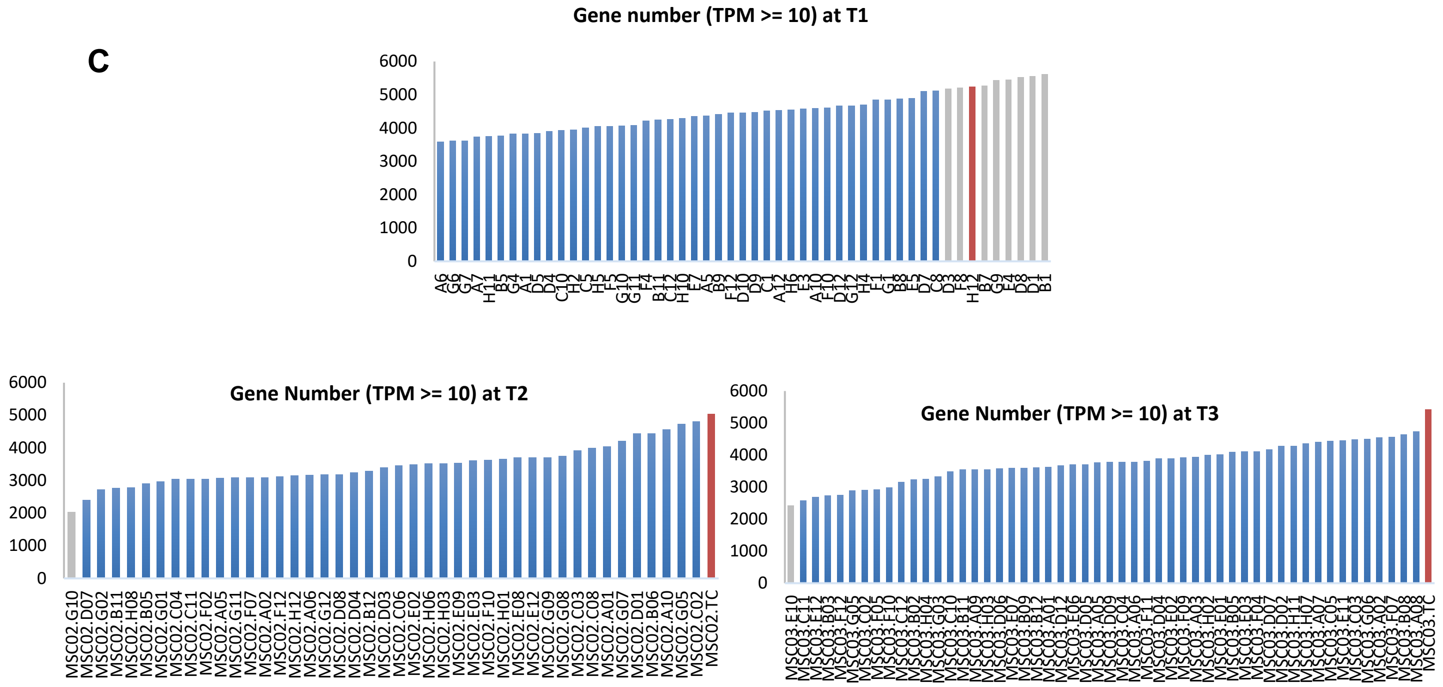

D

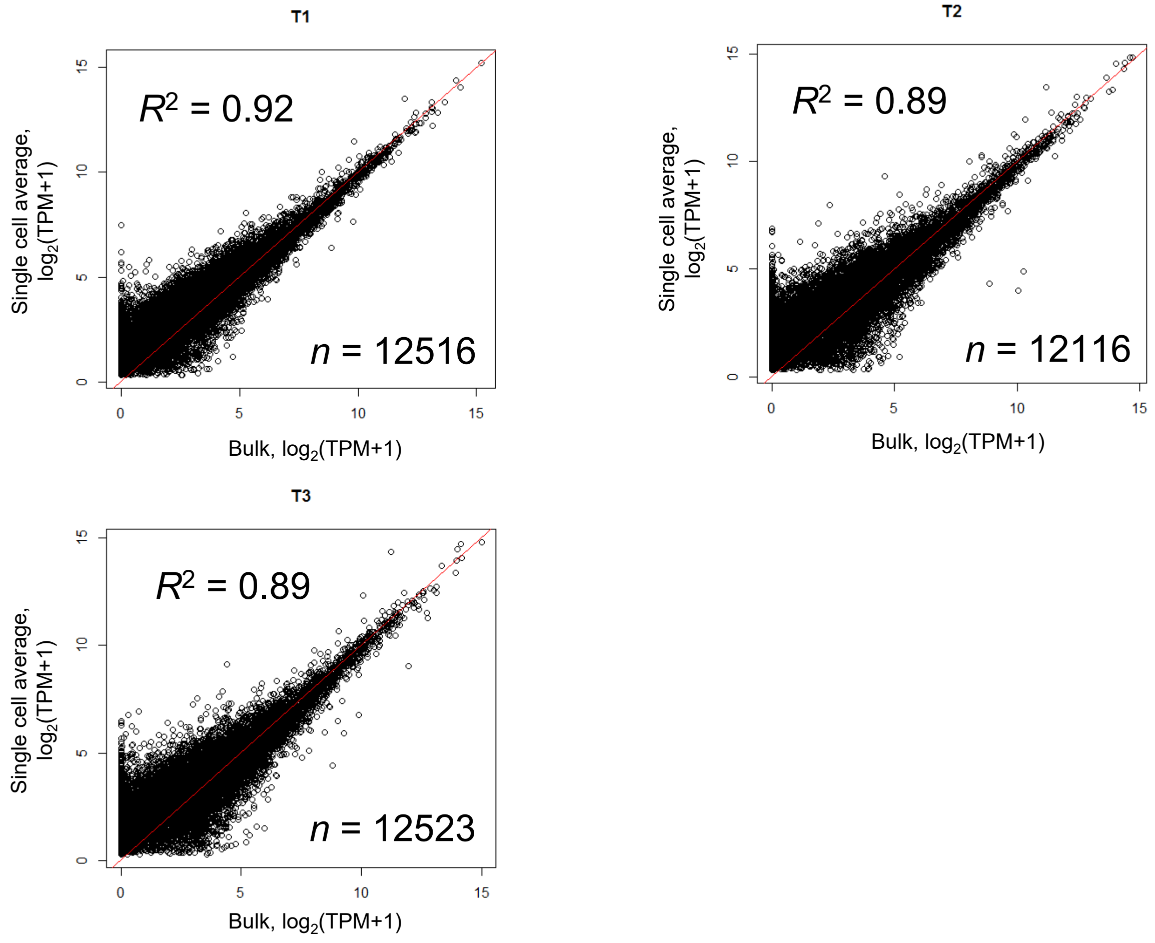

Fig. S2 (Part 3)

E

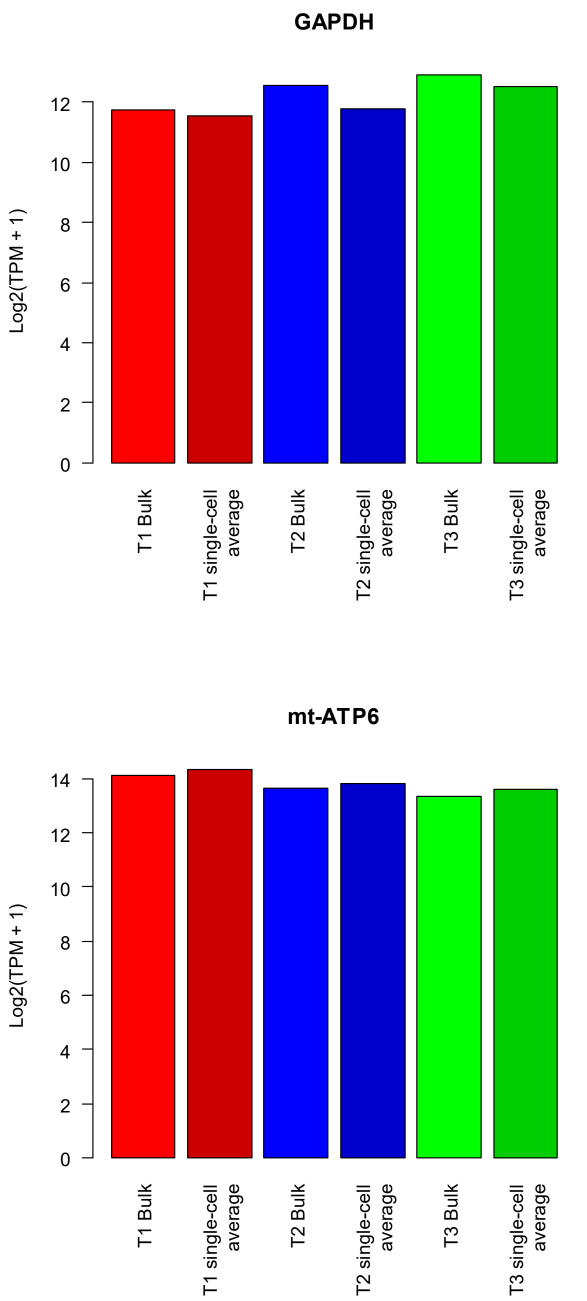

F

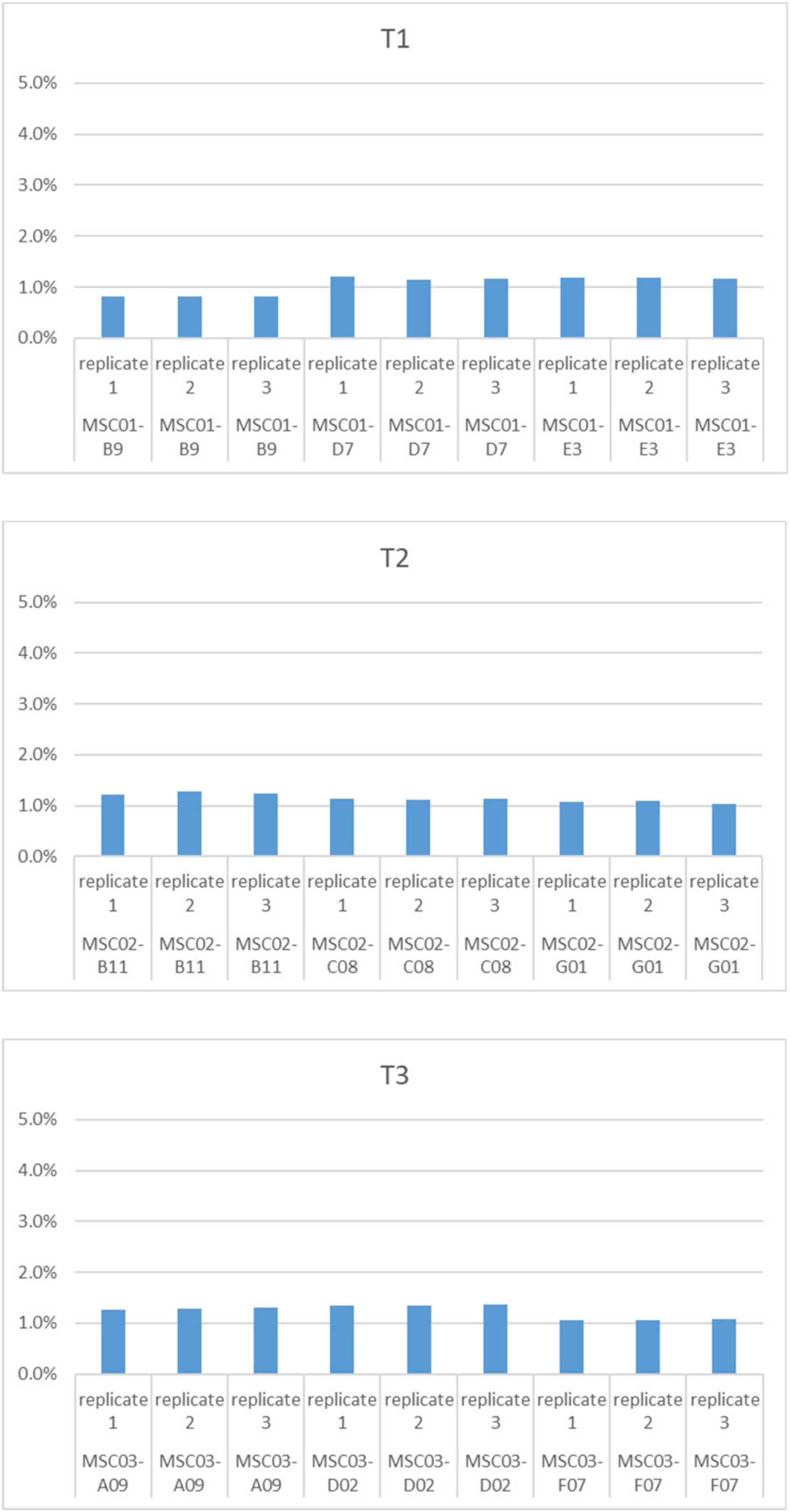

Fig. S3

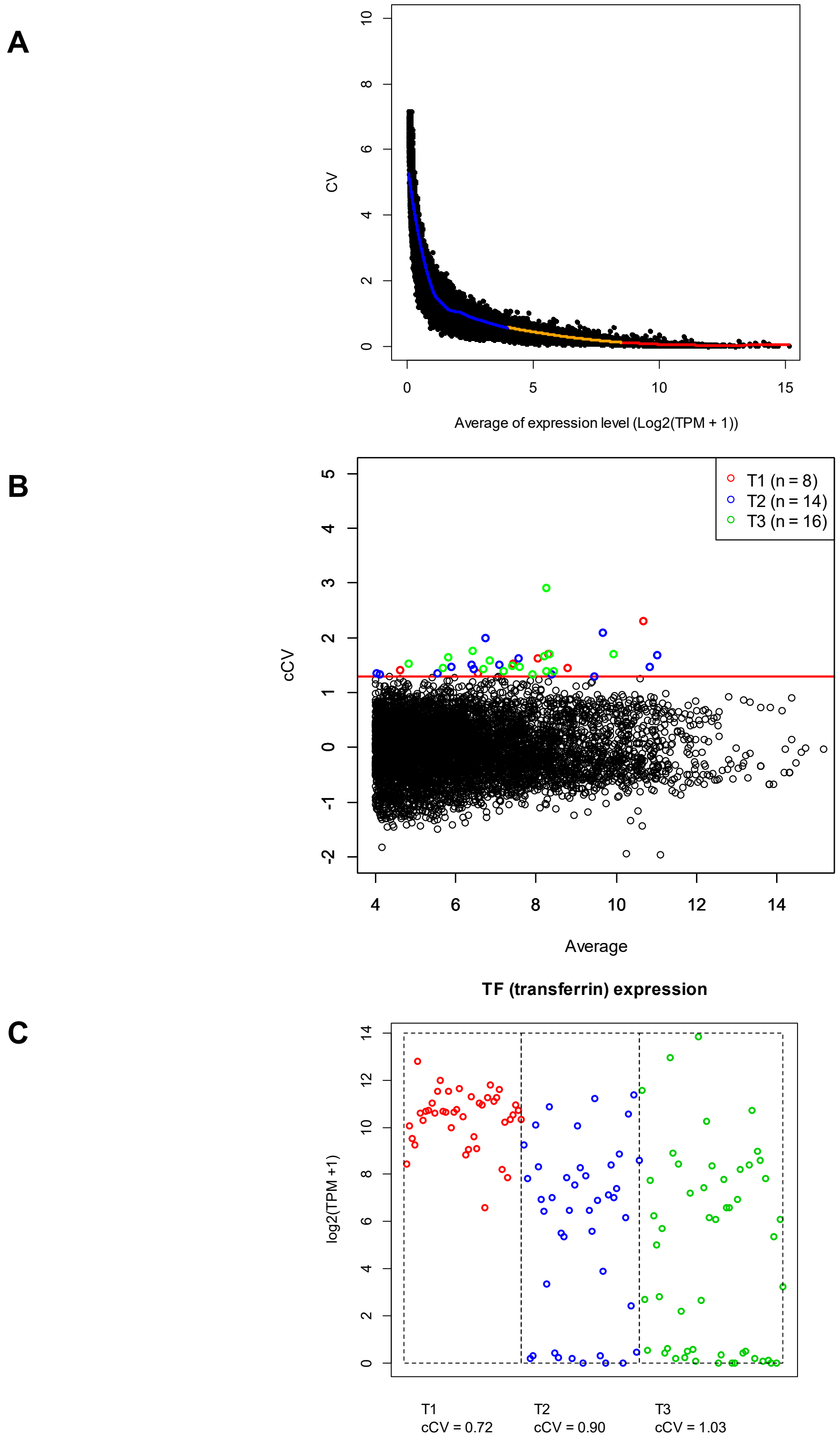

Highly variable genes	T1	T2	T3
ALDOA	1	0	0
B2M	0	1	0
C6orf58	0	0	1
CD9	0	1	0
CLCA1	0	1	1
COPE	0	0	1
COX5B	1	0	0
F3	0	0	1
FAM129B	0	1	0
GSTA1	0	0	1
GSTA5	0	1	0
GUCA2A	0	0	1
GUCA2B	0	1	0
ID2	0	0	1
MGST1	0	1	0
MPC1	0	0	1
MS4A8	0	0	1
OSTC	0	0	1
REG1B	1	0	1
REG3A	0	0	1
REG3A	1	0	1
REG3G	0	1	0
RNF186	0	0	1
S100A6	1	0	0
SDC4	0	1	0
SPTSSB	1	0	0
WFDC2	0	1	0

Fig. S3 c*CV* and highly variable genes. (A) Plot of *CV* versus gene expression levels averaged across single cells. Regression analysis was performed to obtain the locally weighted scatterplot smoothing (LOWESS) curve within the range indicated by each color (blue, n = 30362; yellow, n = 5790; and red, n = 1003). (B) c*CV* and average expression levels. Highly variable genes are shown above the red line and listed in the righthand table, where "1" is assigned when a gene is defined so at the time point; otherwise, "0." (C) c*CV* and distribution of gene expression levels across single cells, illustrated with the transferrin gene. Each circle represents the gene expression level in a single cell.

Fig. S4

A

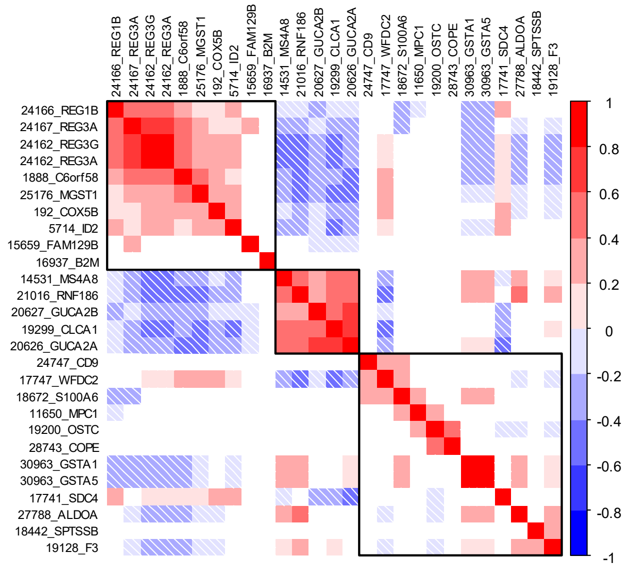

B

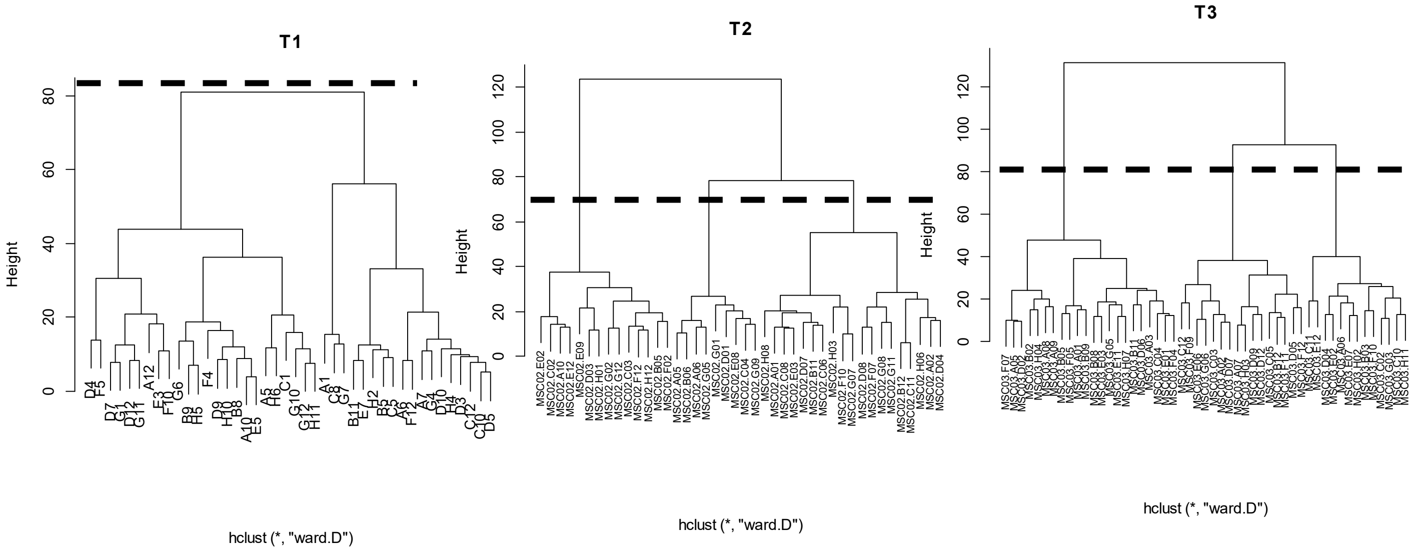

Fig. S4 Determination of gene and cell groups in single-cell RNA sequencing. (A) Correlation plot of highly variable genes. (B) Dendrogram of single cells.

Fig. S5 (Part 1)

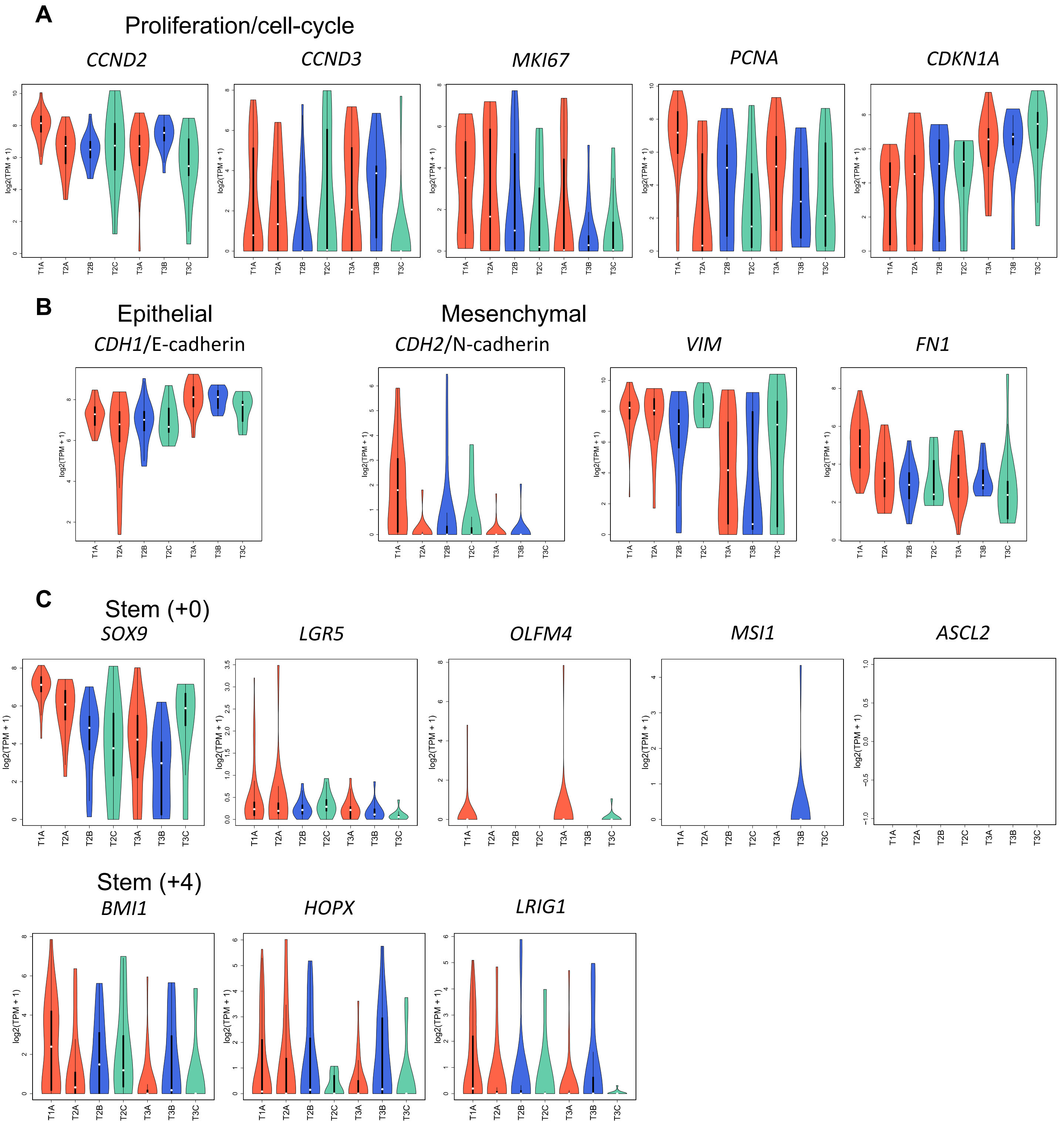

Fig. S5 Violin plots of the expression levels of the marker genes. "A," "B," and "C" following T1/T2/T3 represent the anti-epithelial, cGMP/GC, and dormant cell groups, respectively (*n*: 42 for T1A, 14 for T2A, 19 for T2B, 9 for T2C, 22 for T3A, 16 for T3B, and 13 for T3C). Some genes such as *ASCL2* were not expressed in any category. (A) Proliferation/cell-cycle markers, (B) epithelial and mesenchymal markers, (C) stem cell and differentiation markers, (D) drug efflux markers, and (E) glycolysis markers.

Fig. S5 (Part 2)

Differentiation (Absorption)

CEACAM1 *KRT20* *AQP8* *CA1* *SLC26A3*

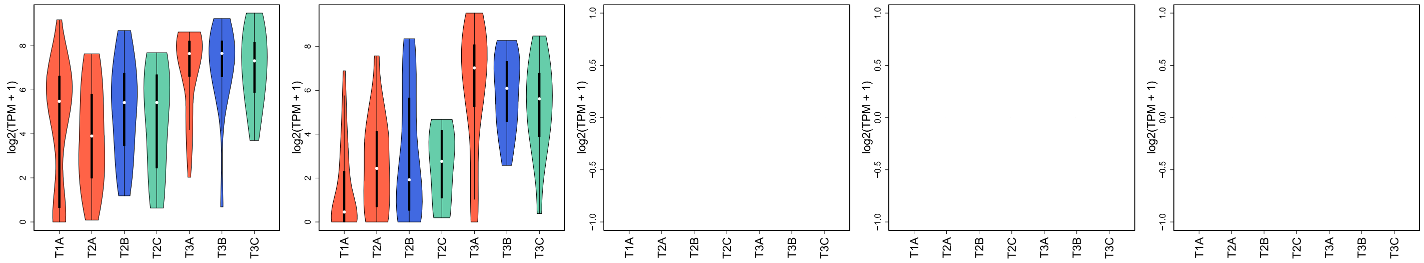

Differentiation (Secretion)

MUC2 *SPINK1*

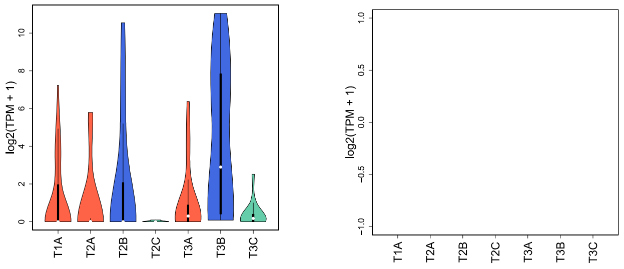

D Drug efflux

ABCB1 *ABCE1*

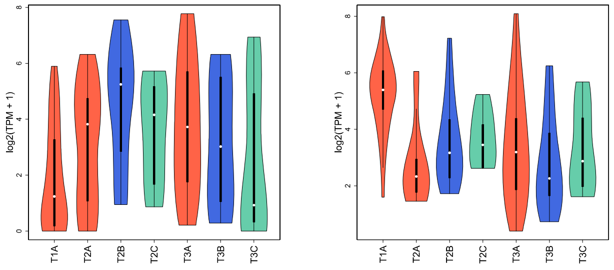

E Glycolysis

PDK1

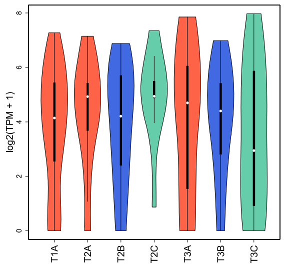

Fig. S6 (Part 1)

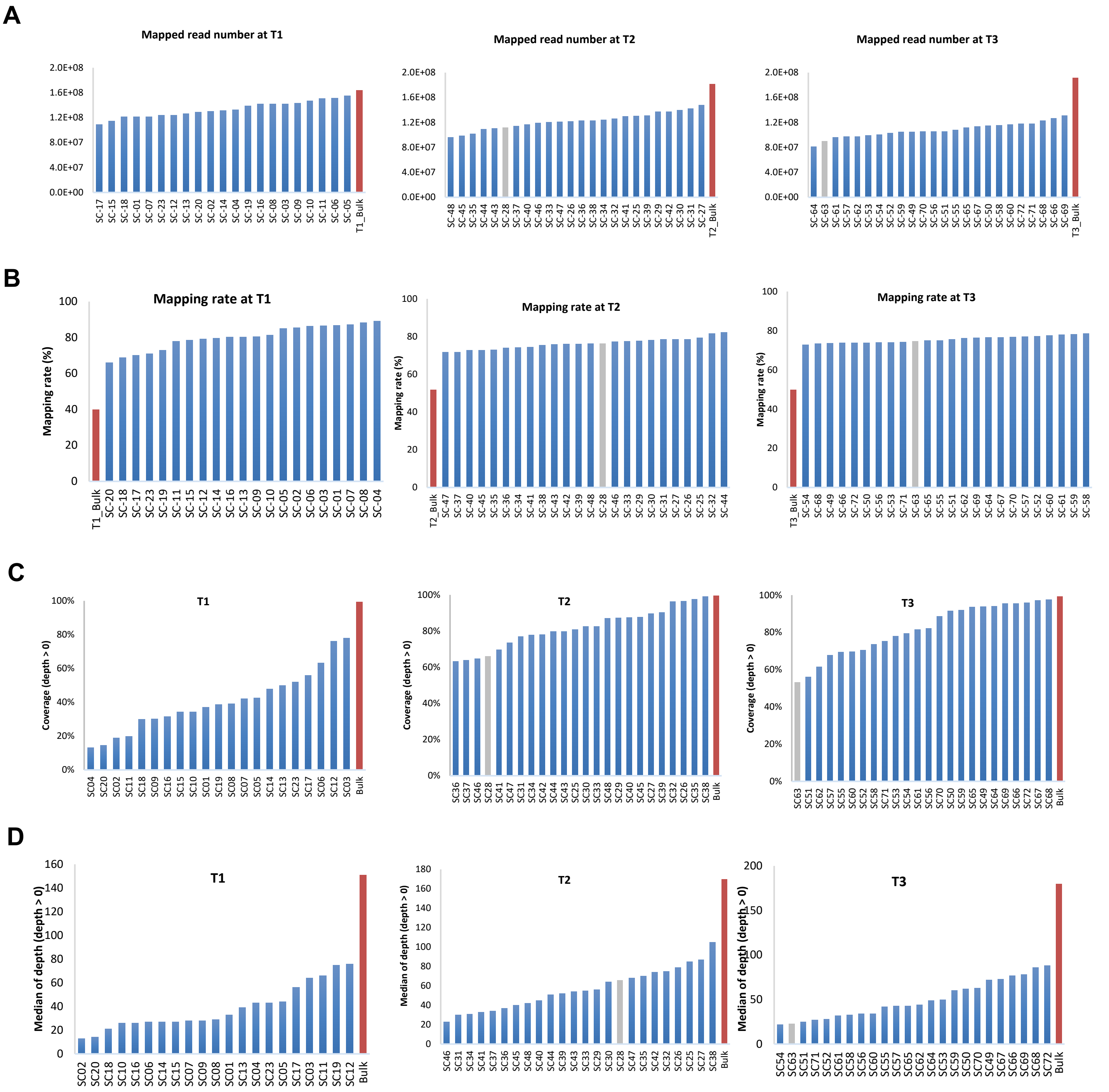

Fig. S6 Quality control check of single-cell exome sequencing data. Bars/curves in red, blue, and gray represent bulk-cell, single-cell, and filtered-out data, respectively. (A) number of mapped reads; (B) mapping rate, (C) coverage of genome with depth > 0, (D) median depth, in which regions with depth = 0 were excluded, (E) Lorenz curve of depth (including regions with depth = 0), (F) Gini coefficients of depth (including regions with depth = 0), (G) ADO rate, and (H) scatter plot of SNVs between VAFs in bulk-cell sequencing and fractions of single cells with SNVs called in single-cell sequencing (*n* at T1, T2, and T3 is 910, 882, and 914, respectively). Black and red lines represent the linear regression and theoretically expected lines, respectively.

Fig. S6 (Part 2)

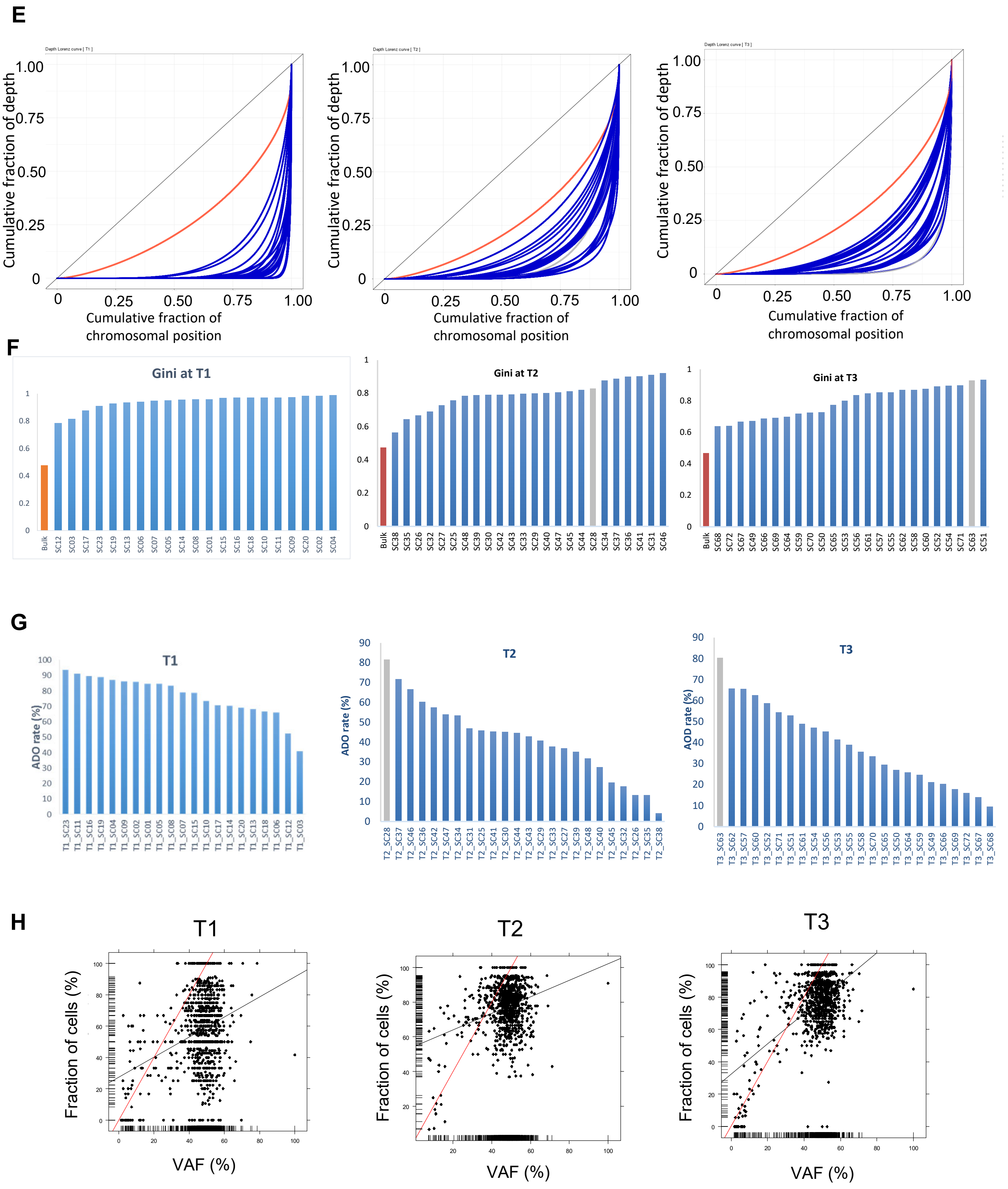

E
Cumulative fraction of depth
Cumulative fraction of chromosomal position
0
0.25
0.50
0.75
1.00
F
Gini at T1
Gini at T2
Gini at T3
Bulk
G
T1
T2
T3
ADO rate (%)
H
T1
T2
T3
Fraction of cells (%)
VAF (%)

Fig. S7

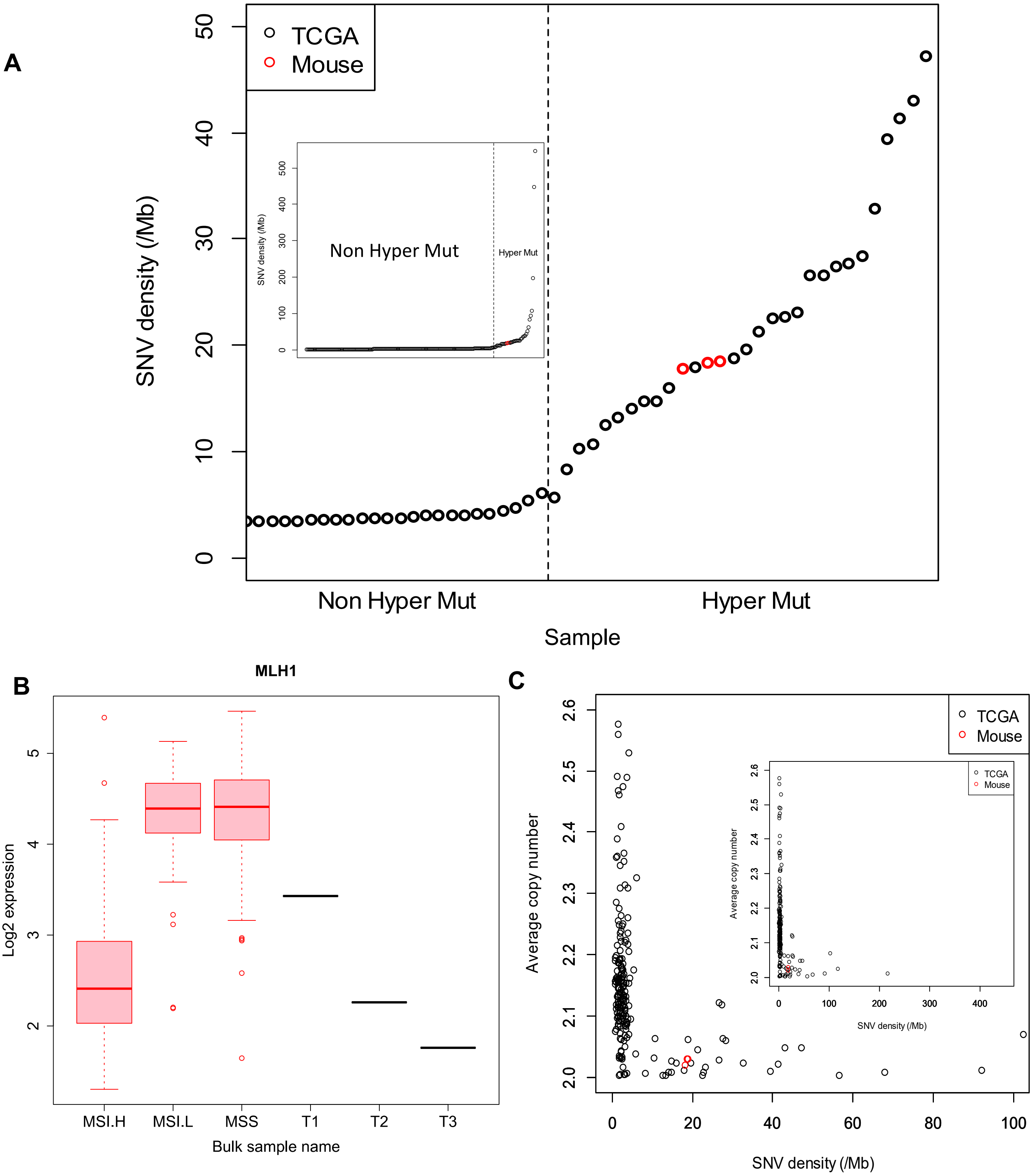

Fig. S7 Association with hypermutation type based on human cancer counterpart to our mouse model. (A) SNV density in human colorectal cancer and in the mouse model. Black and red circles represent TCGA human colorectal cancer samples (n = 244) and mouse samples (n = 3) at T1, T2, and T3, respectively. Dashed lines separate hyper and non-hyper mutation types. (B) *MLH1* expression in TCGA and mouse samples. MSI.H, microsatellite instability high (n = 35); MSI.L, microsatellite instability low (n = 42); MSS, microsatellite stable (n = 166). (C) Average copy number across the genome versus SNV density. Insets in panels A and C show zoomed-out views.

Fig. S8

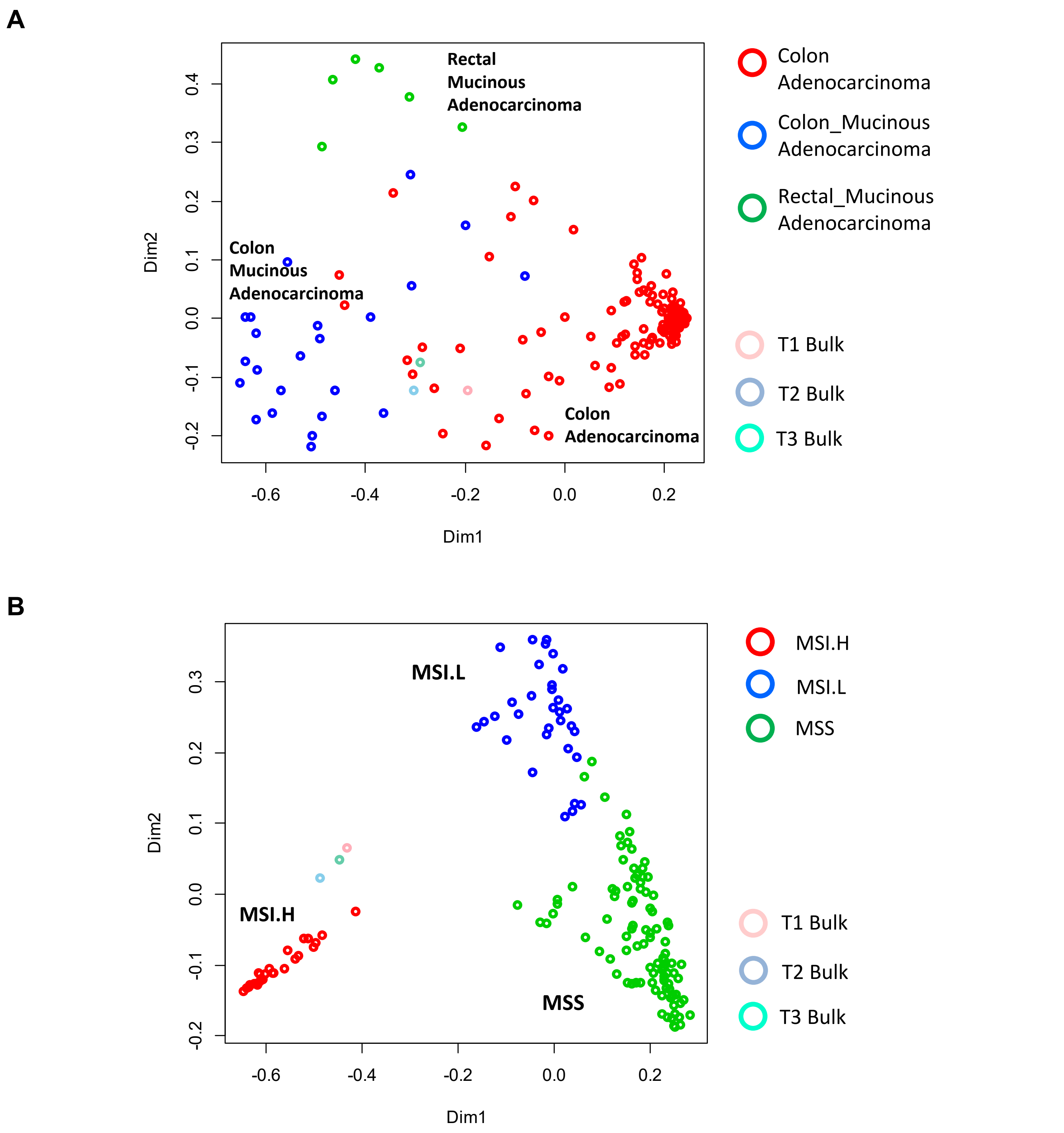

Fig. S8 Associations with histological type and microsatellite instability based on human cancer counterpart to our mouse model. Multidimensional scaling plots generated by Random Forest based on the proximity matrix are shown. (A) For histological type. *n* = 118, 23, and 6 for colon adenocarcinoma, colon mucinous adenocarcinoma, and rectal mucinous adenocarcinoma, respectively. (B) For microsatellite instability. MSI.H, microsatellite instability high (*n* = 35); MSI.L, microsatellite instability low (*n* = 42); MSS, microsatellite stable (*n* = 166).

Fig. S9

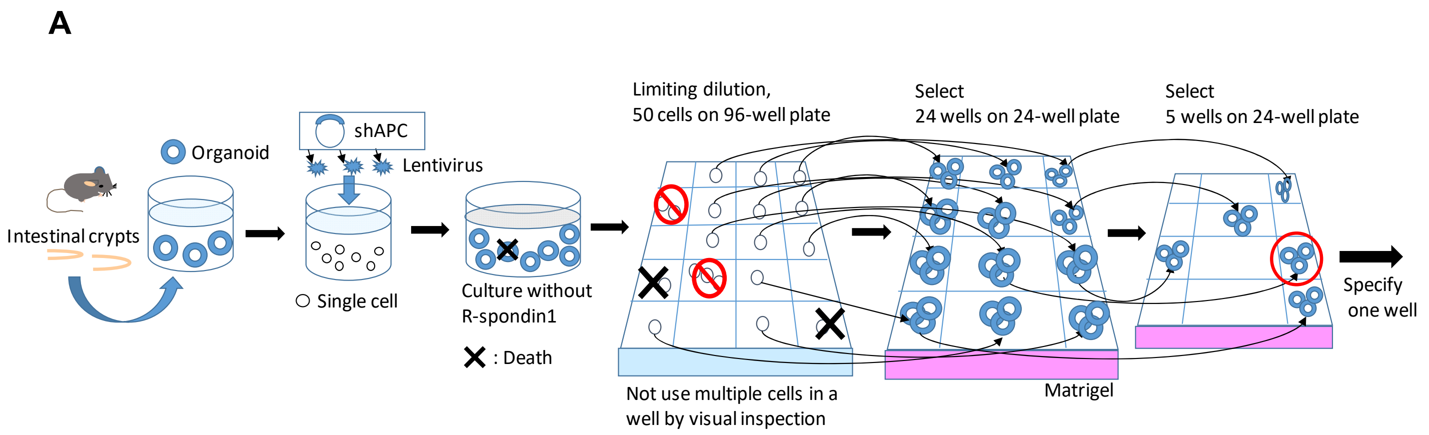

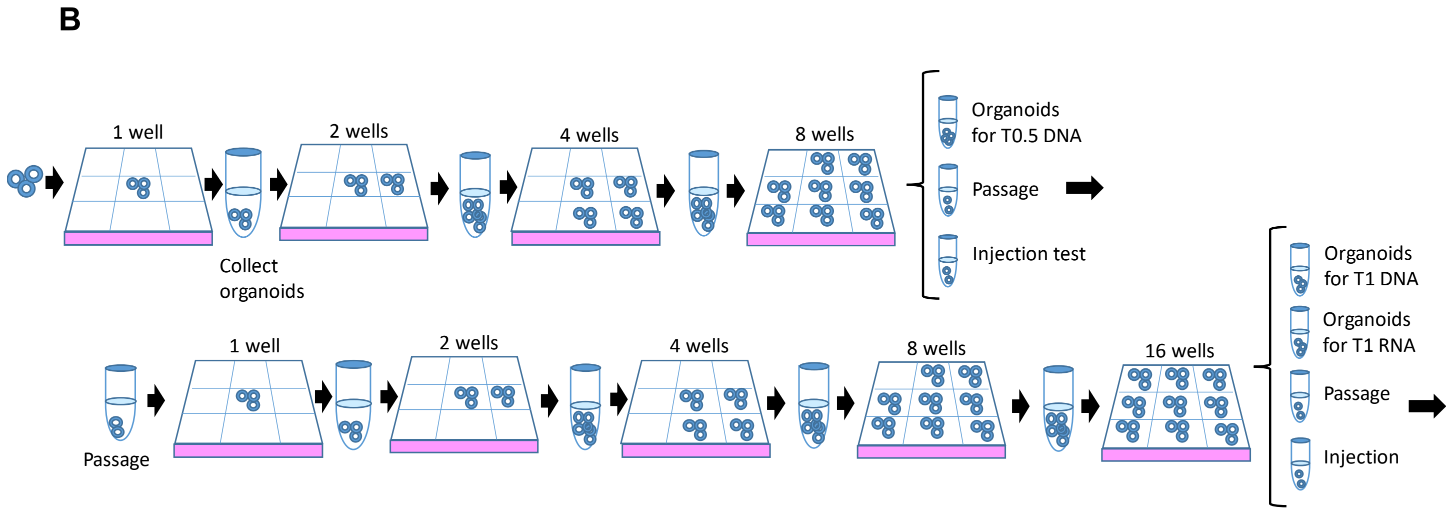

Fig. S9 Schematic representation of the culture experiment. See Materials and Methods for details. (A) Experimental procedure until specifying organoids originating from a single cell. (B) Procedure for the multiplication and sampling of the organoids at T0.5 and T1. The same procedure was used for T2 and T3.

Fig. S10

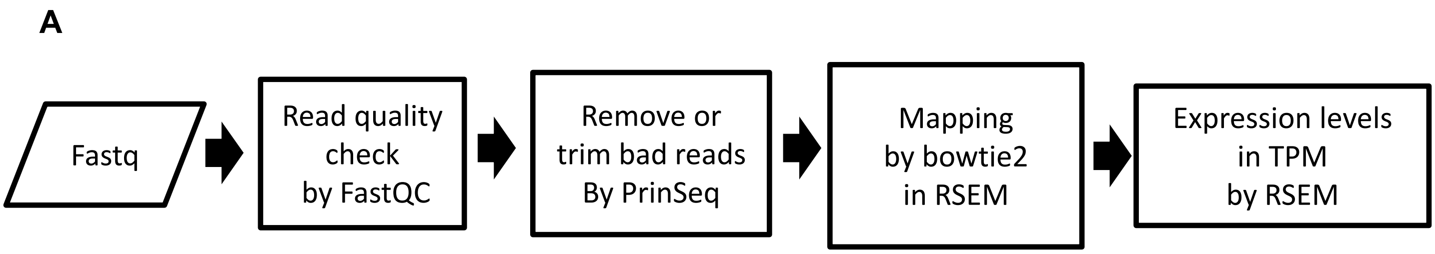

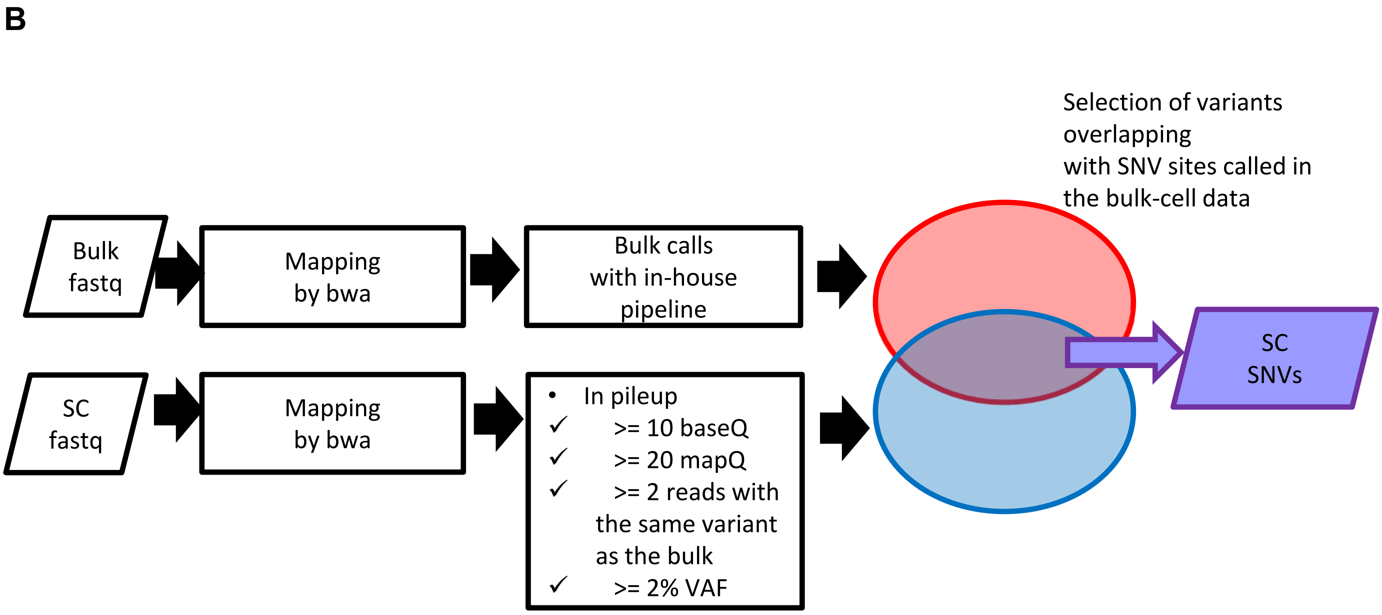

Fig. S10 Procedure for calculating expression levels and for calling SNVs in single-cell sequencing. (A) Procedure for calculating expression levels (TPM). (B) Procedure for calling SNVs in single cells (SCs).
